# Exploiting *Wolbachia* as a Tool for Mosquito-Borne Disease Control: Pursuing Efficacy, Safety, and Sustainability

**DOI:** 10.3390/pathogens14030285

**Published:** 2025-03-14

**Authors:** Riccardo Moretti, Jue Tao Lim, Alvaro Gil Araujo Ferreira, Luigi Ponti, Marta Giovanetti, Chow Jo Yi, Pranav Tewari, Maria Cholvi, Jacob Crawford, Andrew Paul Gutierrez, Stephen L. Dobson, Perran A. Ross

**Affiliations:** 1 Casaccia Research Center, Department for Sustainability, Italian National Agency for New Technologies, Energy, and Sustainable Economic Development (ENEA), 00123 Rome, Italy; riccardo.moretti@enea.it (R.M.); luigi.ponti@enea.it (L.P.); 2 Lee Kong Chian School of Medicine, Nanyang Technological University, Singapore 308232, Singapore; juetao.lim@ntu.edu.sg (J.T.L.); joyi001@e.ntu.edu.sg (C.J.Y.); pranav.tewari@ntu.edu.sg (P.T.); 3 René Rachou Institute, Oswaldo Cruz Foundation, Belo Horizonte 30190-002, Brazil; alvaro.ferreira@fiocruz.br (A.G.A.F.); giovanetti.marta@gmail.com (M.G.); 4 Center for the Analysis of Sustainable Agricultural Systems, Kensington, CA 94707, USA or casas.kensington@gmail.com (A.P.G.); 5 Department of Sciences and Technologies for Sustainable Development and One Health, Università Campus Bio-Medico di Roma, 00128 Roma, Italy; 6 Area of Parasitology, Department of Pharmacy and Pharmaceutical Technology and Parasitology, Faculty of Pharmacy, Universitat de València, 46100 Valencia, Spain; maria.cholvi@uv.es (M.C.); 7 Verily Life Sciences, South San Francisco, CA 94080, USA; jacobcrawford@verily.com (J.C.); 8 Division of Ecosystem Science, College of Natural Resources, University of California, Berkeley, CA 94720, USA; 9 Department of Entomology, University of Kentucky, Lexington, KY 40546, USA or sdobson@mosquitomate.com (S.L.D.); 10 MosquitoMate, Inc., Lexington, KY 40502, USA; 11 Pest and Environmental Adaptation Research Group, School of BioSciences, Bio Molecular Science and Biotechnology Institute, The University of Melbourne, Melbourne, VIC 2052, Australia; perran.ross@unimelb.edu.au (P.A.R.)

**Keywords:** arboviruses, *Wolbachia*, vector control, incompatible insect technique, population replacement strategy, effectiveness, sustainability, safety, community engagement, climate change

## Abstract

Despite the application of control measures, mosquito-borne diseases continue to pose a serious threat to human health. In this context, exploiting *Wolbachia*, a common symbiotic bacterium in insects, may offer effective solutions to suppress vectors or reduce their competence in transmitting several arboviruses. Many *Wolbachia* strains can induce conditional egg sterility, known as cytoplasmic incompatibility (CI), when infected males mate with females that do not harbor the same *Wolbachia* infection. Infected males can be mass-reared and then released to compete with wild males, reducing the likelihood of wild females encountering a fertile mate. Furthermore, certain *Wolbachia* strains can reduce the competence of mosquitoes to transmit several RNA viruses. Through CI, *Wolbachia*-infected individuals can spread within the population, leading to an increased frequency of mosquitoes with a reduced ability to transmit pathogens. Using artificial methods, *Wolbachia* can be horizontally transferred between species, allowing the establishment of various laboratory lines of mosquito vector species that, without any additional treatment, can produce sterilizing males or females with reduced vector competence, which can be used subsequently to replace wild populations. This manuscript reviews the current knowledge in this field, describing the different approaches and evaluating their efficacy, safety, and sustainability. Successes, challenges, and future perspectives are discussed in the context of the current spread of several arboviral diseases, the rise of insecticide resistance in mosquito populations, and the impact of climate change. In this context, we explore the necessity of coordinating efforts among all stakeholders to maximize disease control. We discuss how the involvement of diverse expertise—ranging from new biotechnologies to mechanistic modeling of eco-epidemiological interactions between hosts, vectors, *Wolbachia*, and pathogens—becomes increasingly crucial. This coordination is especially important in light of the added complexity introduced by *Wolbachia* and the ongoing challenges posed by global change.

## 1. Introduction

### 1.1. The Constant Challenge of Vector Control in the Fight Against Arboviral Diseases

Arboviral diseases (i.e., diseases caused by arthropod-borne viruses) represent a major threat to human health due to their impact in terms of loss of lives, reduced quality of life, and the costs associated with preventative measures and treatments [1]. Despite decades of efforts to reduce this burden, arbovirus epidemics are re-emerging in various parts of the world, and emerging in new areas, both in tropical and in more temperate regions [2,3,4,5,6]. This spread is expected to increase with global change [7,8,9,10,11,12,13,14,15,16,17].

Among vectors, mosquitoes play a major role in recent outbreaks of arboviruses, primarily due to their invasiveness, supported by human-aided dispersal [18], and their adaptability to disturbed and urbanized environments [19]. Indeed, mosquitoes are responsible for recent outbreaks of significant diseases like dengue, Zika, chikungunya, yellow fever, West Nile fever, and Rift Valley fever which cause millions of symptomatic cases and more than 700,000 deaths annually [20,21,22,23,24,25]. Additionally, mosquitoes are responsible for other important parasitic diseases, including malaria [26] and filariasis [27]. These health-related problems are coupled with significant economic costs; for instance, the total global cost of dengue alone was estimated at USD 8.9 billion in 2013 [28].

Due to the lack of effective vaccines for most arboviral diseases, vector control remains a high priority for public health [29]. However, the challenge is complex because key mosquito vectors can benefit from climate warming, flooding, deforestation, shorter winters [10] (which might extend the annual transmission seasons), globalization, and urbanization [30]. Furthermore, mosquitoes can rapidly develop resistance to several insecticides [31,32]. This evidence underscores the need for an increased global awareness of the potential risks related to arboviral diseases and highlights the urgency of revising the best available practices for vector control.

### 1.2. Mosquito-Borne Viral Diseases

Mosquito-borne diseases have emerged as a growing global health challenge, with their impact spreading across multiple continents. Arboviruses, such as those in the *Flaviviridae*, *Togaviridae*, and *Bunyaviridae* families, are primarily transmitted by *Aedes* mosquitoes, particularly *Aedes aegypti* and *Ae. albopictus*. Major arboviral diseases include dengue, Zika, chikungunya, and yellow fever, which are responsible for widespread illness and death worldwide. Historically confined to tropical and subtropical regions, these viruses are now expanding their geographic range due to factors such as climate change, rapid urbanization, and global travel, creating significant public health crises in regions like Asia, Africa, South America, and Europe [6,33,34]. Asia remains the epicenter for arboviral transmission, especially for dengue, which accounts for nearly 70% of the global burden [35]. Countries such as India, Indonesia, and the Philippines report millions of cases annually, complicating efforts to control outbreaks due to the interaction of multiple dengue virus serotypes [35]. In Africa, arboviral diseases are also on the rise, with significant outbreaks of dengue, chikungunya, and yellow fever reported in recent years [36,37]. The increasing range of *Aedes* mosquitoes across the continent has led to the emergence of these viruses in areas previously unaffected. For instance, dengue has become a notable health threat in Kenya, Tanzania, and Sudan [38]. Meanwhile, chikungunya has caused widespread outbreaks in East Africa, and yellow fever continues to cause significant morbidity and mortality despite the availability of a vaccine [36]. South America, particularly Brazil, remains a hotbed for arboviral diseases. In 2020, Brazil reported over 2.2 million dengue cases, the highest number in its history [39]. The region also faced a devastating Zika virus outbreak from 2015–2016, which was associated with severe birth defects and had profound social and economic impacts [40]. Chikungunya has similarly established a strong presence in the region, with widespread outbreaks affecting millions across the Caribbean and South America [4]. Europe has seen an alarming expansion of arboviral diseases in recent years, driven by climate change and global travel. Countries such as Italy, France, and Spain have reported locally transmitted cases of dengue and chikungunya, marking a significant shift from the previous occurrence of only imported cases [4,6]. The establishment of *Aedes* mosquitoes, particularly *Ae. albopictus*, in several European countries has facilitated this northward expansion. The global spread of arboviruses is closely linked to climate change and globalization. Rising temperatures are extending the geographic range of *Aedes* mosquitoes, while increased international travel allows viruses to spread rapidly across continents.

### 1.3. Control Methods Against Mosquito Vectors: Evaluating Effectiveness and Sustainability

In the search for additional control tools against mosquito vectors, effectiveness is certainly a fundamental prerequisite, but affordability must be pursued because fighting mosquito-borne diseases requires substantial investments and pluriannual, large-scale programs. The scale of the intervention, its duration, and the necessary coordination efforts and investments exert a determinant impact on the choice of control approaches to be deployed in each specific scenario. Consequently, the evaluation of the effectiveness of a control measure should be coupled with cost-effectiveness studies [41,42].

A large literature on mosquito vector control is available and several reviews have recently summarized the progresses and challenges in this field [43,44,45,46,47]. The above articles highlight how prevention measures such as the use of protective nets, repellents, insecticide-treated clothing and nets, and larval source management, coupled with the use of insecticides against adults and larvae, have been demonstrated to provide short- and medium-term gains and significantly reduce morbidity and mortality from mosquito-borne diseases. However, long-term success would require the integration of these methods with other sustainable solutions that are more suitable for large-scale deployment [48,49] and the development of best practices for integrated management of mosquito vectors based on scientific evidence [50].

This goal can be achieved by defining clear criteria to assess quality, safety, and entomological efficacy of vector control methods and, as a consequence, standardizing methods [51]. The World health Organization (WHO) developed clear guidelines to help in designing appropriate tests to evaluate the effectiveness of mosquito control methods [52]. This process is articulated in a framework of steps and “good research questions” (population, intervention, comparator, and outcome = PICO) aimed at reinforcing the “certainty” derived from the body of evidence obtained from research and systematic reviews [53]. Data that demonstrate epidemiological impact against one or more target diseases can be used to generate WHO recommendations [52].

Well-designed small-scale experimental trials and/or modeling should be performed along with investigations of expected efficacy and cost–benefit analyses to determine the feasibility and benefit to society that can be expected from large-scale deployment [54]. The effects of the control measures on the environment should be taken into consideration seriously because the latter is one of the pillars of sustainable development [55,56,57]. In the context of vector control, negative side effects on the environment and/or non-target organisms (including humans) can affect sustainability. As an example, certain insecticides lead to the presence of toxic residues in food, water, air, and soil [58,59] and a similar threat contributes to the biodiversity crisis [60], affecting beneficial species like pollinators [61,62,63].

Based on the considerations above, innovative control strategies aiming at tackling the global health threat of mosquito-borne diseases should be not only effective but also friendly to the environment and to non-target organisms, because undesired side effects pose risks to their long-term sustainability.

### 1.4. Wolbachia and Its Manipulation for Vector and Disease Control

*Wolbachia* (Rickettsiales: Anaplasmataceae) are common endosymbiotic bacteria of insects, other Arthropoda, and Nematoda which infect host gonads and are vertically inherited through the oocytes [64,65,66]. The presence of these bacteria is often associated with various effects on the hosts’ biology [67], generally favoring the spread of *Wolbachia* infections into heterogeneous populations that include both infected and uninfected individuals [68].

Among the various effects on hosts, *Wolbachia* may induce a phenomenon of reproductive incompatibility, known as cytoplasmic incompatibility (CI), when infected males inseminate females that harbor a different strain of the bacterium or that are uninfected [65,69]. CI results in the early embryonic arrest in incompatible crosses. Toxin–antidote models [70,71,72,73] or host chromatin-modification models [74,75] have been recently developed to explain the mechanism underlying this *post copula* reproductive barrier [76]. Within a species, populations characterized by full or partial reproductive incompatibility may occur naturally. This is the case for *Culex pipiens* [76] and *Drosophila melanogaster* [77] characterized by population-specific *Wolbachia* variants. CI can be unidirectional (Uni-CI) when crosses involve a population harboring a CI-inducing *Wolbachia* strain and an uninfected population. In this case, in the absence of fitness effects associated with the infection [78], the uninfected population is reproductively disadvantaged because uninfected females may produce fertile progeny only when inseminated by uninfected males while infected females can be successfully inseminated by any possible mating. Uni-CI also occurs between two populations sharing certain *Wolbachia* strains in the case that one of them harbors an additional incompatible *Wolbachia* strain. Similarly to the previous case, females carrying the additional *Wolbachia* strain are reproductively advantaged and, because of this, the spread of the multiple *Wolbachia* infection is favored [79,80] unless a negative effect on fitness is associated with this infection type [79]. In the case of fitness costs and any maternal leakage, *Wolbachia* is only favored when it reaches a threshold frequency in the population where, below this point, the advantage of CI is not enough to favor spread [81].

Differently, CI can be bidirectional (Bi-CI) in the case of crosses between individuals carrying reciprocally incompatible *Wolbachia* strains. This scenario is characterized by fertile crosses between individuals carrying the same infection and incompatible crosses when infected males inseminate females that harbor a different *Wolbachia* strain. In the absence of differences in the fitness of the two populations (determined by *Wolbachia* or by the specific genetic background) and assuming complete maternal transmission of the infection, none of them is reproductively advantaged and the chance of fixation of a single infection type only depends on the frequency of the individuals carrying that infection [82].

**Figure 1:**
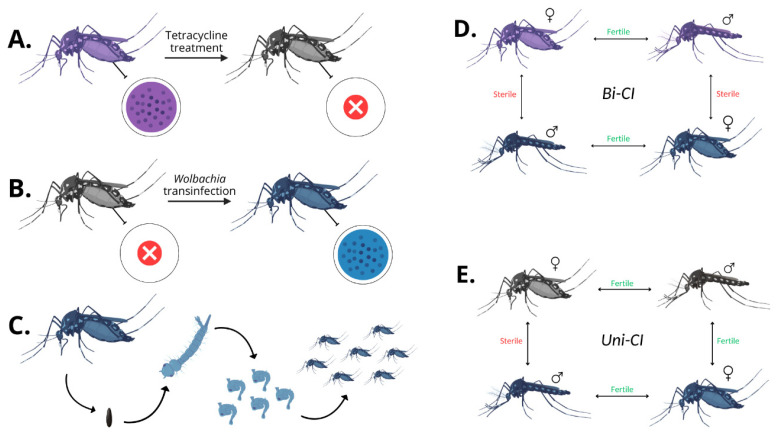
*Wolbachia* transinfection and possible cytoplasmic incompatibility (CI) patterns: (**A**) *Wolbachia* transinfection can be preceded by an antibiotic treatment to cure the native (purple) *Wolbachia* infection; (**B**) *Wolbachia* transinfection can be conducted in naturally uninfected species, in *Wolbachia*-cured populations, or by adding a further *Wolbachia* strain to the native infection; (**C**) In suitable species, *Wolbachia* is transmitted vertically via the maternal cytoplasm; (**D**) A bidirectional CI pattern (Bi-CI) characterizes two different populations of the same species infected by reciprocally incompatible *Wolbachia* strains; (**E**) A unidirectional CI pattern (Uni-CI) characterizes crosses between a population harboring an incompatible *Wolbachia* strain (blue) and a population lacking this strain (gray), regardless of the fact that the two populations may share further *Wolbachia* strains.

The capacity to artificially introduce *Wolbachia* in a target species (*Wolbachia* transinfection) (Figure 1) [83] has led to the exploitation of artificially induced CI as a control tool based on the release of incompatible males to reduce the fertility of wild populations (incompatible insect technique = IIT) [65]. This objective can be pursued by various strategies: establishing a CI-inducing *Wolbachia* infection in species that are not infected naturally [84,85,86,87,88], replacing the native *Wolbachia* with other foreign strains [89,90,91,92], or introducing additional foreign strains to those that are already present in the species [79]. For an overview of the results of these strategies, see Table 1.

Certain *Wolbachia* strains have also been demonstrated to reduce the vector competence of mosquitoes for several key arboviruses, both when these bacteria are naturally present and when they are artificially introduced in the species [93,94] (Table 1). Pathogen inhibition (PI) mainly regards viruses with a positive-sense single-stranded RNA genome ((+)RNA) and has been explained through mechanisms of perturbations to lipid and cholesterol transport mediated by *Wolbachia*, leading to a competition with viruses for essential resources [67,95,96]. However, a contribution to the phenomenon by the capacity of these bacteria to produce reactive oxygen species (ROS) and to modulate host cell autophagy has also been hypothesized [95]. These mechanisms may vary between *Wolbachia* strains and hosts and in some cases are positively correlated with the *Wolbachia* density in the host midgut [95]. *Wolbachia* was also found to suppress *Plasmodium falciparum* infection in a transinfected line of *An. stephensi* by regulating several immune genes [97].

**Table 1:** *Wolbachia* transinfections in mosquito vectors and main induced effects. Only in vivo studies are reported ^a^.

Mosquito Host	*Wolbachia* Strain	Transinfection Method ^b^	Desirable Traits for Vector or Disease Control ^c^	Stability of the Infection and Fitness Effects on Host If Any ^d,e^	Blocked Pathogens
*Ae. aegypti*	*w*Mel	*Wolbachia* microinjection in wild-type embryos [79,85,98]; *Wolbachia* introgression [99,100,101]	Uni-CI, PI	Stable infection; costs to fecundity, fertility, and quiescent egg viability [85,102]; high temperatures during preimaginal stages increase these negative effects but can also lead to a decrease in *Wolbachia* density [102,103,104,105,106,107]; CI leakage when males are obtained from eggs stored for a long time [107]	DENV [85,87,108,109,110,111,112,113,114,115,116,117,118,119,120,121,122,123,124,125,126], ZIKV [87,127,128,129,130], CHIKV [130,131,132], YFV [131,133], MAYV [134,135], SFV [87], KUNV [109]
	*w*AlbB	*Wolbachia* microinjection in wild-type embryos [68,86,135,136]; *Wolbachia* introgression [137,138]	Uni-CI, PI	Stable infection [136,139]; costs to fertility, longevity, and quiescent egg viability [107]; high temperatures associated with long-term egg storage can further reduce egg viability, female fecundity, and *Wolbachia* density [87,107]	DENV [87,117,140,141,142,143], ZIKV [87], SFV [87]
	*w*AlbA	*Wolbachia* microinjection in wild-type embryos [79,87]	Uni-CI, PI	Stable infection; costs to longevity and quiescent egg viability [87]	ZIKV [144]
	*w*Au	*Wolbachia* microinjection in wild-type embryos [87]	PI	Stable infection; costs to longevity and quiescent egg viability	DENV [87], ZIKV [87], SFV [87]
	*w*MelPop	*Wolbachia* microinjection in wild-type embryos [84]	Uni-CI, PI	Stable infection; substantial costs to longevity, egg fertility, and other traits [84,145,146,147,148]; CI, *Wolbachia* density, and vertical inheritance of the infection affected by heat stress during preimaginal stages [149]	DENV [85,113,116,124,150,151], CHIKV [150], YFV [131]
	*w*MelCS	*Wolbachia* microinjection in wild-type embryos [152]	Uni-CI, PI	Stable infection; costs to fertility and quiescent egg viability	DENV [117,152]
	*w*MelM	*Wolbachia* microinjection in wild-type embryos [98]	Uni-CI, PI	Stable infection; costs to fertility and quiescent egg viability	DENV [98]
	*w*Pip	*Wolbachia* microinjection in wild-type embryos [152]	Uni-CI	Stable infection; costs to fertility, longevity, and quiescent egg viability	No effects against DENV and KUNV [109]
	*w*Ri	*Wolbachia* microinjection in wild-type embryos [152]	Uni-CI, PI	Stable infection	DENV [152]
	*w*AlbA + *w*AlbB	*Wolbachia* microinjection in adult females [153]; *Wolbachia* microinjection in wild-type embryos [79]	Uni-CI	Stable infection [79]; imperfect vertical inheritance [153]	No data
	*w*Au + *w*AlbB	*Wolbachia* microinjection in wild-type embryos [87]	Uni-CI	No data	No data
	*w*Mel + *w*AlbA	*Wolbachia* microinjection in wild-type embryos [79]	No data	No data	No data
	*w*Mel + *w*AlbB	*Wolbachia* microinjection in wild-type embryos [79,116]	Uni-CI, PI	Reduced longevity and egg hatching compared to uninfected and *w*Mel- *w*AlbB- single-infected lines [116]	DENV [116]
	*w*Mel + *w*AlbA + *w*AlbB	*Wolbachia* microinjection in wild-type embryos [79]	Uni-CI	Unstable infection; self-CI, displacement of *w*AlbA *Wolbachia* from the ovaries	No data
*Ae. albopictus*	*w*Pip	*Wolbachia* microinjection in *Wolbachia*-cured wild-type embryos [89,154]	Bi-CI, PI	Stable infection	ZIKV [155]
	*w*Mel	*Wolbachia* microinjection in *Wolbachia*-cured wild-type embryos [90]	Bi-CI, PI	Stable infection; sensitive to high temperatures during preimaginal stages	DENV [90], CHIKV [156]
	*w*MelPop	*Wolbachia* microinjection in *Wolbachia*-cured wild-type embryos [91]	Bi-CI, PI	Stable infection; costs to longevity	No data
	*w*Ri	*Wolbachia* microinjection in *Wolbachia*-cured wild-type embryos [157]	Bi-CI (incomplete)	Imperfect maternal transmission, partial self CI	No data
	*w*Riversi	*Wolbachia* microinjection in *Wolbachia*-cured wild-type embryos [158]	Uni-CI	No data	No data
	*w*Pip + *w*Mel	*Wolbachia* microinjection in *Wolbachia*-cured wild-type embryos [155]	Bi-CI; PI	Stable infection	DENV [155], ZIKV [155], CHIKV [155]
	*w*AlbA + *w*AlbB + *w*Au	*Wolbachia* microinjection in wild-type embryos [159]	PI	Moderate fitness effects	DENV [159], ZIKV [159]
	*w*AlbA + *w*AlbB + *w*Ri	*Wolbachia* microinjection in wild-type embryos [160]	Uni-CI	Stable infection	No data
	*w*AlbA + *w*AlbB + *w*Pip	*Wolbachia* microinjection in wild-type embryos [161]	Uni-CI; PI	Stable infection	DENV [161], ZIKV [161]
	*w*AlbA + *w*AlbB + *w*Mel	*Wolbachia* microinjection in wild-type embryos [79]	Uni-CI	Stable infection; self-CI, displacement of *w*AlbA *Wolbachia* from the ovaries	No data
	*w*AlbA + *w*AlbB + *w*MelPop	*Wolbachia* microinjection in wild-type embryos [162]	Uni-CI (incomplete)	Maternal inheritance affected by blood type; costs to fecundity, fertility, and longevity	No data
*Ae. polynesiensis*	*w*Riversi	*Wolbachia* introgression [163]	Uni-CI	Stable infection	No data
	*w*AlbB	*Wolbachia* microinjection in *Wolbachia*-cured wild-type embryos [164,165]	Uni-CI; PI	Stable infection	DENV [164], *Brugia pahangi* [166]
*Ae. vexans*	*w*AlbB	*Wolbachia* microinjection in wild-type adults [86]	No data	Unstable infection	No data
*An. stephensi*		*Wolbachia* microinjection in wild-type embryos [88]	Uni-CI	Stable infection	*Plasmodium falciparum* [88,97]
*Cx. quinquefasciatus*	*w*AlbB	*Wolbachia* microinjection in *Wolbachia*-cured wild-type embryos [92,167]	Bi-CI	Stable infection	No effects on *Plasmodium relictum* [167]
	*w*Pip + *w*AlbA	*Wolbachia* microinjection in wild-type embryos [92]	Uni-CI	Stable infection	No data

Uni-CI = Unidirectional Cytoplasmic Incompatibility; Bi-CI: Bidirectional Cytoplasmic Incompatibility; PI = Pathogen Interference. ^a^ References regard studies conducted on both laboratory-reared and field-collected individuals while studies conducted on cell lines are excluded; this latter information is instead included in Ant et al. [95]; ^b^ Processes of *Wolbachia* introgression are also included, even if this approach to establishing new host–endosymbiont associations is not based on *Wolbachia* microinjection but rather on the gradual replacement of the genetic background of the host; ^c^ References related to the CI pattern can be found in the column describing the establishment of the infection while references related to PI are listed in the column showing the blocked pathogens; the shown CI pattern is relative to the crosses with the wild-type individuals of the species; ^d^ For stable *Wolbachia* infection we mean full, or almost full, vertical transmission and sufficiently stable *Wolbachia* density under natural environmental conditions; ^e^ Specific references regarding the effects of the infection are only reported in the case of more than one article describing the specific transinfection or highlighting specifically an effect, otherwise this information is available from the article cited in the third column.

**Figure 2:**
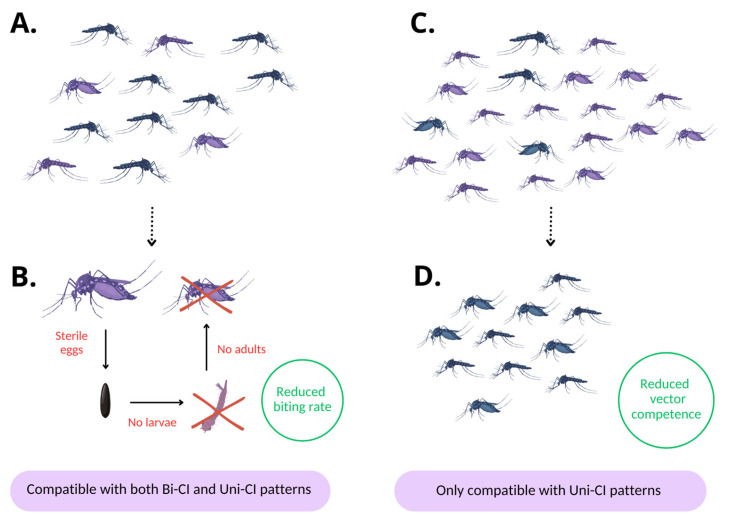
*Wolbachia*-based control strategies (IIT vs. PRS). (**A**,**B**): Incompatible insect technique (IIT): (**A**) Incompatible males (blue) are released to outnumber wild males (purple) and reduce the chance by a wild female to encounter a fertile mating; (**B**) The aim of the strategy is to strongly reduce the number of females capable of reproducing, leading to a suppression of the wild population and to a reduction of the biting rate. (**C**,**D**) Population replacement strategy (PRS): (**C**) Males and females of a mosquito population characterized by a *Wolbachia* infection inducing a Uni-CI pattern and a reduced vector competence are concurrently released to replace the wild-type population; (**D**) As the released population spreads (due to CI), the frequency of the females with reduced vector competence increases and the transmission of the arboviruses by mosquitoes decreases.

PI by *Wolbachia* has enabled new control programs not aimed at mosquito elimination but based on the replacement of wild-type populations with populations of the same species with a reduced capability to transmit arboviruses (Figure 2) [65,100,168]. This strategy (population replacement strategy = PRS) is only feasible when Uni-CI patterns occur, and releases necessarily also involve mosquito females. The process of replacement is supported by the CI phenomenon which, as already explained, favors infected over uninfected females [169]. This process can become self-sustaining above a threshold frequency that is determined by eventual differences regarding fitness associated with the *Wolbachia* infection or with the genetic background of the released compared to the autochthonous individuals [170].

One additional strategy for *Wolbachia*-based vector control involves the use of genes derived from these bacteria to transform species that are poorly suitable for a stable infection. This is the case for *Anopheles gambiae*, in which no stable transinfections have been generated, but CI has been induced through the introduction of the genes that are responsible for this phenomenon in natural hosts [171]. *Ae. aegypti* has also been the target of this approach [172] and may provide an alternative to *Wolbachia* infections in some environments.

All of the above control strategies belong to a group of control methods which we refer to as genetic control strategies (GCSs) [173,174] and which share common advantages and issues (Section 2.1). In this context, the choice of a defined *Wolbachia*-based control strategy and the opportune *Wolbachia* strain to be used for this objective must be made based on the target vector species, the pathogens which this species may transmit, and its *Wolbachia* infection type. This selection should also consider the initial necessary investments and prospects of long-term viability, taking into account the environmental context, potential evolutionary changes, regulations related to the exploitation of these bacteria, and the possible concerns by the community [175].

Herein, we review IIT and PRS basics and applications, highlighting all the steps that have brought these strategies to open field deployment and concluding by discussing certain specific issues and the perspectives on implementation.

## 2. The Exploitation of *Wolbachia* for Disease Control: A Practical Guide to Open Field Deployment

### 2.1. Genetic Control Strategies: Potential and Practical Issues with a Specific Focus on *Wolbachia*-Based Strategies

GCSs targeting mosquito-borne diseases are based on the production and release of modified mosquitoes to achieve either vector population suppression, by an autocidal approach, or vector population modification, through the spread of heritable traits reducing pathogen transmission [173,174,176] (Table A1). The modifications mentioned above can be achieved through irradiation or chemical treatments applied exclusively to the individuals intended for release. Alternatively, they can be implemented by permanently altering a specific line of the target species using genetic approaches or by introducing specific endosymbiotic bacteria to harness their effects on the host [65]. GCSs are highly specific because the action of the modified mosquitoes is necessarily limited to individuals of the same species. Furthermore, modifications are generally considered safer for the environment compared to insecticides as they are not based on the field release of any active molecule or dangerous organism except individuals of the same species that is targeted. However, concerns from the public about potential environmental impacts persist in the case of control strategies involving genetically modified organisms (GMOs). Therefore, there are several countries where GCSs based on GMOs are not permitted. The regulation of the use of symbiotic bacteria is still debated in certain countries (see Section 2.3), however, their open field deployment is generally more easily allowed because it does not involve genetic modifications.

**Figure 3:**
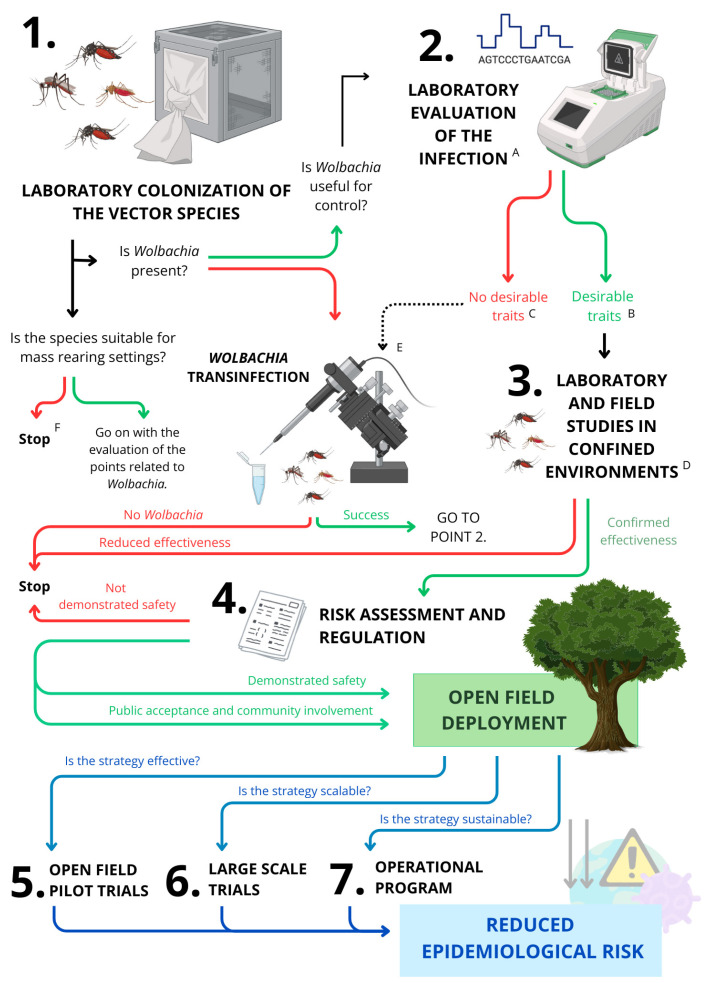
Steps for the deployment of *Wolbachia*-based control strategies and possible issues. Green arrows indicate a positive result and red arrows a negative result. ^A^ Laboratory evaluation aimed at investigating the stability of the infection and the effects on host; ^B^ In the case of populations within the same species showing differential vector competence or reciprocal CI due to differences in *Wolbachia* infection type; ^C^ In the case of homogeneous *Wolbachia* infections within the same species; ^D^ Experiments to test the possibility of exploiting *Wolbachia* to control the wild population or reduce its vector competence; ^E^ In the case of the presence of a native infection with no desirable traits for disease control, two possible transinfection strategies can be attempted: (i) removal of the native *Wolbachia* infection and replacement with alternative *Wolbachia* strains chosen based on the expected effect (CI or CI and PI); (ii) addition of an opportune *Wolbachia* strain to the native infection to enable the laboratory population of the vector to express CI or CI and PI; ^F^ Species showing poor suitability for mass rearing settings are generally unsuitable for all genetic control strategies.

Considerations on effectiveness, safety, and sustainability guide the evaluation of any GCS and can determine at different stages the interruption of programs aimed at open field deployment. Figure 3 schematizes this process regarding *Wolbachia*-based control strategies and subsequent sections will examine the key factors that could determine a positive or a negative outcome.

#### 2.1.1. Vector Population Suppression: Pros and Cons

The suppression of vector populations can be pursued by releasing, in a defined area, large numbers of males that compete with wild males to inseminate females but, due to an opportune modification, cause them to be unfertile. In the case of mosquito vectors, males are relatively harmless because they are not known to bite or transmit pathogens that cause human diseases (Table A1).

Male infertility can be achieved by treating males with ionizing radiation (or more rarely with chemicals) at a dose capable of inducing sterility without significantly affecting their fitness (sterile insect technique = SIT; [177,178,179,180] or exploiting natural phenomena of reproductive incompatibility such as those associated with the presence of the endosymbiotic bacterium *Wolbachia* (Section 2.1, IIT; [65]). A combination of SIT and IIT has also been tested as a strategy to control *Aedes* species [138,161]. For the same purpose, laboratory lines genetically modified to only produce sterile males [181,182,183] or engineered to carry a dominant lethal genetic system (release of insects carrying a dominant lethal = RIDL; [184]) have also been developed. Recently, an *Ae. aegypti* line engineered with a tetracycline-off genetic switch to cause complete female lethality in early larval development has also been established and field tested [185]. As previously mentioned, another genetic control approach proposed to control *Ae. aegypti* is based on the transgenic expression of the genes from *Wolbachia* determining CI to recapitulate this phenomenon without involving transinfection [172]. A CRISPR–Cas9 gene drive system targeting female reproduction has also been developed and tested at laboratory scale [186].

The reproductive potential of the target population can be strongly affected by autocidal approaches because the probability of a female encountering a fertile mating is reduced with an increased proportion of sterile males among total males, leading to a decline in the growth rate of the population and, therefore, in the number of vectors across generations [187]. A reduced number of vectors results in reduced pathogen transmission. This implies that a strong decrease in the risk of disease outbreaks can be achieved even without eradication in the case that a specific threshold of vector density (specific to the vector, to the pathogen, and to the area) is not reached [188,189,190].

Despite potential advantages over other control strategies in terms of effectiveness, specificity, and eco-compatibility, genetic control methods aimed at mosquito suppression possess certain constraints that can impede large-scale applications (Table A1) [173,174]. These issues relate to: (i) the suitability of the species for the approaches; (ii) the quality of the mass reared population compared to the wild population; (iii) challenges with sex separation; (iv) the overall sustainability of the program.

Not all target species are equally suitable for colonization in the laboratory and may not be amenable to the mass rearing conditions required to produce large numbers of males in a short time. Selection under mass rearing conditions acts to improve their performance under artificial rearing conditions but can lead to an impoverishment of genetic variability which can be detrimental under open field conditions [191,192,193]. Additionally, wild-type populations are generally better adapted to the local environmental conditions and can be characterized by the acquisition of mutations inducing resistance to certain insecticides [170,194]. These factors may result in a lower male mating competitiveness of the released individuals compared to the wild-types due to reduced survival or flight ability, that should be ascertained, case by case, to better evaluate the potential of the control strategy [195]. The periodical outcrossing of the laboratory line with a sufficient number of wild individuals can be a means to preserve genetic variability and to also maintain genotypes that are resistant to certain insecticides [196].

Focusing on IIT, not all insect species are amenable to *Wolbachia* infection and, as an obvious consequence, unsuitable vectors cannot be targeted by this control strategy [86,197]. Furthermore, certain fitness effects associated with *Wolbachia* infection can reduce the efficiency of mass rearing or the ability to store infected eggs for a long time [136,198,199,200] (Table 1). The ability to outcross *Wolbachia*-transinfected populations depends on the *Wolbachia* infection type. Outcrossing is always possible when Uni-CI patterns occur with wild-types because wild-type males are fully fertile with all female types (Section 1.4). In the case of Bi-CI patterns, wild-type males generally induce egg inviability when crossed with females harboring a different *Wolbachia* infection type. However, this issue can be addressed by curing the *Wolbachia* infection in wild-caught individuals before crosses or by exploiting reduced levels of fertility that may occur when CI is not complete or when it is reduced by a factor as like male aging [201,202].

In addition to problems related to the reduction of the genetic variability, the quality of males to be released can be affected by the mass rearing conditions, by the sterilizing treatment (when needed, as in the case of SIT), and by the procedures of packaging, temporary storage, and delivery of the males prior to release [203,204].

The need for an efficient sexing procedure is another major issue common to all strategies based on the release of sterile males, mainly because even low percentages of residual females could translate to thousands of individuals when millions of males are released, and these females could increase the biting rate locally [205,206]. In the case of IIT, releasing females with an artificial *Wolbachia* infection may cause a further issue, i.e., the undesired spread of the naïve infection that could prevent further suppression by releases of the same strain (because a population carrying the same *Wolbachia* infection of released males would no longer be incompatible with this strain) [207]. This issue is more likely to occur in IIT programs based on Uni-CI patterns (as in the case of *Wolbachia*-infected *Ae. aegypti*) because contaminant females would be fertile when mating with any male. This risk is somewhat lower in the case Bi-CI patterns because released females would be sterilized by wild males that would be present at increasing frequency proportionally to the distance from the treated area and as a consequence of their migration from the surroundings [208,209].

In *Aedes* and *Culex* mosquitoes, sexing protocols can take advantage of protandry and smaller mean size of male pupae to sort the individuals at this stage through metal sieving plates or Fay–Morlan glass separators and can be at least partially automated [205,210,211]. These mechanical methods cannot ensure the complete absence of females, but their frequency can be reduced to less than 1% [205,211]. Furthermore, mechanical sexing becomes less efficient as the number of managed larvae increases, that is typical of large-scale programs [212]. However, recent advances in automated sex sorting at the pupal stage seem promising [213].

Sexing procedures can also exploit certain genetic markers that are naturally present in mosquito vector species or can be introduced by transgenesis [214,215]. As an example, laboratory lines expressing fluorescence can be separated by complex object parametric analyzer and sorter (COPAS) sorting [216]. As a potential support to sterile insect technique, genetic sexing strains (GSSs) have been developed for various mosquito species, including *Ae. aegypti* [217] and *Ae. albopictus* [154]. Self-sexing strains have also been developed through the exploitation of a repressible gene determining female lethality [185]. A similar effect has also been achieved through RNAi [218], but significant challenges remain to be solved before large-scale deployment is feasible [219].

Artificial intelligence (AI)-based technology has also been applied to *Ae. aegypti* for sexing freshly emerged adults and is capable of reducing the female contamination rate to approximately 1 in 900 million [220].

Regarding sustainability, it is worth noting that suppressing a vector population through the release of sterile males is a self-limiting control strategy as it depends on the continuous production and release of individuals unless pest or vector eradication is achieved [221]. The possibility of success depends on the quantity and timing of mosquito releases, with more frequent releases over a longer period and with more individuals expected to have greater effects on the target vector population. Additionally, the benefits of the control program are expected to last for longer when the treated area is larger due to dispersal of the released males and immigration of wild-type mosquitoes from the surrounding areas, that generally occur at the borders of the treated area [222]. These limitations make autocidal control-based strategies more suitable for area-wide application than small-scale deployment.

Another important parameter to be considered when planning sterile male releases for vector suppression, and essential for measuring their success, is the rate of released males relative to wild males because, as the ratio increases, the chance of a female to mate successfully diminishes [178]. Consequently, both effectiveness and costs for male production and delivery are expected to increase with this ratio. However, as the distribution of mosquito populations is spatially and temporally not homogeneous [223], evaluating this parameter becomes increasingly difficult and less worthwhile as the scale of the releases increases.

One final limitation of sterile-male-based control strategies against mosquito vectors is that, unlike some agricultural pests, the populations of these vectors are generally regulated by density-dependent factors mostly acting during the larval stage because breeding sites and/or nutrients are often limited. Therefore, population suppression will tend to be offset by reduced density-dependent mortality [224]. RIDL based on a late acting dominant lethal has been suggested has a means to avoid this problem because lethality occurs in mature larvae that have already competed for resources [184]. In other cases, this factor should be taken into consideration when evaluating the potential of genetic control.

#### 2.1.2. Vector Population Modification: Pros and Cons

An alternative strategy to vector suppression is the use of gene drive systems [225,226] that may exploit nuclear genes, genes carried by transposons, or heritable endosymbionts (including *Wolbachia*) to reduce the vectorial capacity of a target population [174,221,227,228,229,230,231] (Table A1). The success of this strategy relies on the partial or complete replacement of wild individuals with ones carrying genes or endosymbionts which reduce pathogen transmission. Vector population modification also requires a mechanism to induce a deviation from normal Mendelian inheritance to bias the inheritance of the desired phenotype [221]. This modification can be self-sustaining once reaching a threshold in the population [81], which depends on the mode and fidelity of transmission bias and the host fitness costs of the modification [100,225].

As already discussed, CI has been theorized and implemented to support the spread of *Wolbachia* strains causing a reduction in lifespan or vector competence of wild vector populations, both effects leading to a reduction in the vectorial capacity (see Section 1.4 with regard to PRS). Numerous *Wolbachia* transinfections have now been generated (Table 1) but only a small number have been released for vector population modification (Section 2.1 and Section 2.5.2; Table 2). Several genetic constructs in *Ae. aegypti* have also been engineered to reduce mosquito vector competence [232,233,234,235] or provide a drive mechanism [236,237] but are yet to have been released under field conditions.

Mosquito–*Wolbachia* associations for PRS require extensive laboratory trials to ascertain their invasive potential and stability and impact on vector competence. An ideal *Wolbachia* strain for this approach would provide complete or strong arbovirus transmission blocking, possess perfect transmission fidelity and a strong drive mechanism, and remain stable under a range of environmental conditions with minimal impacts on host fitness.

A PRS has two key advantages over approaches relying on suppression, including incompatible and sterile releases but also more traditional control. First, there is an expectation that fewer mosquitoes will be required to achieve stable population modification than substantial suppression from incompatible or sterile releases, and the lack of a requirement for sex sorting means that production is relatively straightforward. Second, if stable population modification is achieved the approach can be, as previously mentioned, self-sustaining, with no need for ongoing releases to maintain suppression of arbovirus transmission. In the long term, PRS releases, if the modification is stably maintained, are expected to provide substantial cost savings compared to other approaches which rely on temporary suppression [221].

Releases for PRS not only rely on the production, release, and quality control of the insect strain but also extensive community engagement to ensure public awareness and approval of the releases [238,239]. PRS will cause a temporary increase in the number of biting females in the mosquito population which may seem counterintuitive and be perceived negatively by the public. Genetic constructs that modify characteristics of the mosquito population may also be perceived less favorably than genetic constructs which aim to suppress populations [239]. One other potential limitation of population modification is the risk that successful population modification will not have the desired effect on arbovirus transmission. Unlike suppression, where a population reduction will reduce arbovirus transmission risk, there is no guarantee that the modified population will have reduced vectorial capacity under field conditions. However, to date, all published estimates of *Wolbachia* replacement programs indicate substantial suppression of dengue (Section 2.5.2).

Finally, vector population modification releases (at least for those based on endosymbionts) are subject to environmental conditions to a greater extent than population suppression releases [149,170,240].

Factors affecting PRS success are complex and may include the choice of *Wolbachia* strain, the quality and quantity of the released mosquitoes, the size of the release zone [241], genetics of the released and target populations [170], climatic factors [104,242], and aspects of the built environment which influence mosquito reproduction and dispersal [243]. There may be some contexts where *Wolbachia* replacement is unachievable, for instance, where the host fitness costs of *Wolbachia* infection are too high relative to the fidelity of CI and maternal transmission. For instance, a *w*MelPop infection introduced in *Ae. aegypti* has failed to persist under field conditions despite reaching near-fixation, likely due to substantial host fitness costs [145,244].

### 2.2. Safety of *Wolbachia*-Based Control Strategies

The release of mosquitoes with manipulated *Wolbachia* infections into the field raises potential safety concerns, including undesired evolutionary changes, horizontal transmission of *Wolbachia* to other species, an increase in the abundance of non-target vectors, and other potential ecological impacts. These issues have been investigated by several research groups and public agencies worldwide before open field trials [245,246,247,248,249]. Overall, the risks related to the exploitation of *Wolbachia* have been evaluated as negligible, but not all concerns can be addressed through laboratory experiments. The widespread release of *Wolbachia* strains around the world has provided an opportunity to further evaluate these safety concerns.

There are potential risks associated with the release of *Wolbachia* mosquito strains that do not occur naturally in the local population, including horizontal transmission of *Wolbachia* to other species and the introduction of foreign genetic material. While the potential for horizontal transmission of transinfections has been experimentally tested [246] and considered unlikely, no surveys have been performed following field releases of *Wolbachia* to test for the presence of the transinfection in non-target species such as predatory insects or those that occupy a similar ecological niche. Although the specific *Wolbachia* strains used for releases are not typically present naturally in the target species, *Wolbachia* occur in approximately 50% of insect species, as well as other arthropods and in several species of nematodes throughout the world [250]. Insects with a widespread distribution like *D. melanogaster*, *Ae. albopictus*, and *Cx. pipiens* are already infected with *Wolbachia* and the *Wolbachia* strains generally used for mosquito control are isolated from these same species. Humans frequently interact with insects with *Wolbachia* and releasing insects carrying *Wolbachia* strains already common in nature is unlikely to pose any additional threat. As an obligate endosymbiont, *Wolbachia* cannot reproduce outside host cells or persist in the environment in the long term or infect other organisms through the production of spores [251]. *Wolbachia* bacteria are not compatible with vertebrate physiology [252] and there is no evidence that *Wolbachia* releases pose a direct risk to humans or other vertebrates.

The establishment of a *Wolbachia* transinfection in a natural population may introduce mitochondrial and nuclear alleles which were not previously present [253,254], with an increasing likelihood for a mosquito strain which is genetically distinct from the target population. While backcrossing can be used to increase the genetic similarity between the release strain and the target population [170,201], this may not be feasible for releases over large areas with high genetic diversity. Concerns have recently been raised that the widespread release of a single homogenous *Wolbachia*-infected population across ecologically diverse regions could lead to the spread of undesirable traits [255], though genomic studies suggest that mosquito populations can still remain genetically distinct despite population replacement by the same release stock [254]. There is also limited evidence that *Wolbachia* infections themselves will have any undesirable impacts, with strains being released showing robust virus blocking [95] and no impacts of *Wolbachia* on insecticide resistance [256].

Once established at a high frequency, the population replacement of mosquitoes with *Wolbachia* transinfections may be difficult to reverse, which could make future interventions more challenging [257]. The establishment of the *w*AlbB strain in an *Ae. aegypti* population carrying *w*Mel, for instance, is much less likely to succeed than establishment in an uninfected population due to crosses between strains resulting in bidirectional incompatibility (Section 1.4). While achieving stable, high frequencies of *Wolbachia* is the intended objective of population replacement programs, reversibility may be desirable if the establishment of *Wolbachia* has undesired effects such as viral enhancement or becomes less effective at suppressing arbovirus transmission over time [257]. While some studies have demonstrated increased virus densities or transmission due to *Wolbachia* infection, an overwhelming number of studies show that the strains released for population replacement in *Ae. aegypti* show blocking [95], though there is still potential for environmental conditions to weaken effects of *Wolbachia* on virus blocking [103]. Selection on viral resistance to the blocking effects of *Wolbachia* might be expected to occur in the long term, but there is no evidence of viruses evolving to bypass *Wolbachia* at this stage [258]. While phenotypic changes in the expression of *Wolbachia* effects have been documented in experimental host populations maintained both with and without deliberate selection pressures, these evolutionary changes are due to nuclear changes and not *Wolbachia* changes, with *w*Mel and *w*AlbB *Wolbachia* genomes remaining unchanged following transinfection and field release [259,260,261,262].

To reduce risks of releasing strains with poor performance, strains should be tested extensively in the local context. This includes testing vector competence against currently circulating isolates and testing mosquito traits under local environmental conditions. Forecasting impacts of evolutionary changes will also require long-term monitoring of *Wolbachia*, viruses, and mosquito genomes and phenotypes.

Similar to traditional control methods, mosquito release programs run the risk of impacts on non-target species and the broader ecosystem. An increase in non-target species abundance is a potential outcome of both population replacement and especially suppression releases targeting a single species (including *Wolbachia*-based IIT but also SIT and gene drives). For replacement releases, populations with a high frequency of *Wolbachia* could now have lower fitness due to direct fitness costs of the *Wolbachia* strain, as well as potential self-incompatibility and CI. For suppression releases, this is expected to reduce the population size of the target species directly. In both cases, this could facilitate invasion of non-target species if they overlap in their ecological niche. In Yogyakarta, Indonesia, the establishment of *w*Mel in *Ae. aegypti* had a minimal increase in relative abundance [263] but impacts may be larger for suppression releases where target populations will be suppressed to a greater extent. In Singapore, the suppression of *Ae. aegypti* through incompatible male release led to an overall increased abundance of *Ae. albopictus*, but this increase was heterogenous across release sites, with a substantial increase in some and no increase in others [264]. In this case, suppression of *Ae. aegypti* and an increase in *Ae. albopictus* is unlikely to be a problem for overall dengue suppression but may be an issue to be evaluated carefully in locations where *Ae. albopictus* is an important vector.

Few other ecological impacts beyond mosquitoes have been explored and more work is needed to understand potential impacts on the broader ecosystem, though these are unlikely to be greater than with a reliance on non-specific insecticides.

### 2.3. Legal Framework Related to the Use of *Wolbachia* as a Vector Control Tool

Open field deployment of a new control tool generally requires risk assessment studies [245,246,265,266,267] that are conducted following procedures specific to each country or supranational organization. Based on the different regulatory frameworks, *Wolbachia* and the insect carrying *Wolbachia* can be classified differently, and this classification determines the possibility of using *Wolbachia* for experimental or operational purposes [268]. Specific authorizations are generally needed before biocidal products like *Wolbachia* can be made available on the market (as an example, see [269]).

The first country where the use of mosquitoes with *Wolbachia* has been regulated is the United States, through the intervention of the U.S. Environmental Protection Agency (EPA), which is under the Federal Insecticide, Fungicide and Rodenticide Act (FIFRA). *Wolbachia*-transinfected strains were assimilated into biopesticides in 2017 and, before any release is permitted, an Experimental Use Permit (EUP) is required to be issued by the EPA, in addition to approval by local authorities [270]. As an example, following successful pilot demonstrations (see Table 2), both *Ae. albopictus* (ZAP males) and *Ae. aegypti* (WB1 males) were granted Section 3 registration as public health pesticides [271]. Currently, additional state and local approvals are being pursued, with the intent to use *Wolbachia*-induced CI to suppress these important mosquito vector populations in the USA.

In Australia, *Wolbachia* is classified as a substance by the Pesticides and Veterinary Medicines Authority and *Wolbachia*-transinfected insects are evaluated as veterinary chemical products [265]. The regulation is established based on the Agricultural and Veterinary Chemicals Code Act 1994, which defines the requirements and processes for the evaluation of the safety and efficacy of these products, and the environmental regulations under the Environmental Protection and Biodiversity Conservation Act 1999, which considers potential impacts on biodiversity and the environment.

In Singapore, the deployment of *Wolbachia*-transinfected mosquitoes is a government-led initiative primarily overseen by the National Environment Agency (NEA), operating under the Ministry of Sustainability and the Environment, titled Project *Wolbachia* [272]. The NEA conducts continual risk assessments and quality assurance of the transinfected mosquitoes prior to any release. This government-led approach ensures that public health and environmental safety are held paramount, with the NEA coordinating efforts across various stakeholders and maintaining transparency and accountability in the program’s implementation.

In Europe, Regulation No. 528/2012 of the European Parliament and of the Council concerning the placing on the market and use of biocidal products defines the term biocidal product and establishes the rules for its use and placing on the market in the EU. In Annex V of that Regulation *Wolbachia* is framed in Product Type 18, relating to insecticides, acaricides, and products to control other arthropods. A further Commission Implementing Decision (EU) 2018/1623 of 29 October 2018 [273] specified that bacteria of the genus *Wolbachia* or preparations containing such bacteria used to inoculate mosquitoes with the aim of creating artificially infected mosquitoes for vector control purposes shall be considered biocidal products within the meaning of Article 3(1)(a) of Regulation (EU) No 528/2012. Instead, artificially infected mosquitoes, irrespective of the infection technique used, shall not be considered biocidal products or treated articles.

In Latin America, the Pan American Health Organization (PAHO) is the main body in charge of coordinating actions in response to the current dengue health emergency. A Vector Control Advisory Group (VCAG) validated the impact of the PRS program conducted by the World Mosquito Program against *Ae. aegypti* on the reduction of dengue cases [274]. Although there is no clear legislation on the matter, VCAG, within its evaluation of innovative strategies for *Aedes* control, does qualify the infrastructure and planning necessary to carry out an action that implies the use of *Wolbachia* [275]. However, the approval process is conducted based on agreements and regulations at a national level. As an example, to ensure compliance with national regulations, the program introduction of *w*Mel-infected *Ae. aegypti* in Brazil underwent an approval process with key regulatory bodies, including the National Health Surveillance Agency (Anvisa) (No. 216/2022/SEI/DIRE3/ANVISA [276]), the Brazilian Institute of the Environment and Renewable Natural Resources (Ibama) (No. 02001.003179/2013-03 [277]), and the Ministry of Agriculture, Livestock and Food Supply (MAPA) (No. 21000.005439/2013-21 [278]). Authorization was also obtained from the National Research Ethics Commission (CONEP) [279].

### 2.4. Public Acceptance and Initiatives to Favor Community Engagement

A clear regulation is also expected to favor the implementation of *Wolbachia*-based control programs by promoting transparent and understandable communication, preventing social alarm and allowing the population to trust in the goal and collaborate for it. Engagement of local communities is fundamental for supporting the efforts of public and private organizations to control mosquito vectors [238,280]. This importance has been highlighted by the Global Vector Control Response 2017–2030 strategy of the World Health Organization (WHO) [281] that considers the development of plans for the effective engagement and mobilization of communities in vector control among the priority activities to be pursued. As an example, the PRS programs run in South American countries were supported by strategies of awareness of the population. Specifically, to achieve community acceptance, the World Mosquito Program (WMP) developed the Public Acceptance Model, an ethical methodology so that people can understand the work that is being carried out, as well as be able to participate in it, obtain answers to their doubts voluntarily, and decide on the infected mosquito releases in their neighborhood and city [282]. The activities primarily targeted public schools, health units, and social leaders, encouraging discussions on the link between health, the environment, and arbovirus control. Teachers were trained to integrate the *Wolbachia* method into educational activities, empowering communities to critically assess the intervention [238]. Also, in Vietnam, the WMP has been operating since 2006 in Nha Trang with oversight by the Ministry of Health and started on 2020 in southern Vietnam with high rates of public acceptance with approximately 4500 public surveys performed by the WMP [283].

Similarly, Project *Wolbachia* Singapore was supported by a communication campaign including a comprehensive Q&A section where the population can resolve the main doubts about issues related to the *Wolbachia* technique, leading to a high acceptance of the intervention by citizens [284]. The NEA worked to demystify *Wolbachia* by providing accessible educational materials that explained the scientific concepts behind the technology, such as the natural occurrence of *Wolbachia* bacteria and how releasing male *Wolbachia*-infected mosquitoes reduces *Ae. aegypti* populations. Scientists and technicians actively engaged with the community through door-to-door visits, dialogue sessions, and roadshows, offering hands-on experiences like interacting with male mosquitoes to demonstrate they do not bite. Educational initiatives were also conducted in schools and childcare centers to involve younger audiences, who could further convey information to their families. These efforts resulted in high public awareness and acceptance, with surveys indicating that over 90% of residents had no concerns about mosquito releases in their neighborhoods [284,285,286].

### 2.5. Main *Wolbachia*-Based Programs of Disease Control Worldwide and Their Results

#### 2.5.1. Incompatible Insect Technique

The first IIT trials date back to the 1960s when a local population of *Cx. pipiens fatigans* (*Cx. quinquefasciatus*) was eliminated through the release of incompatible males [287], even though at the time *Wolbachia* was not known as the CI causative agent. However, despite the reported field success, the work was limited only to areas in which naturally occurring *Wolbachia* incompatibilities could be identified, and as a result, the applied use of *Wolbachia* was not extended for more than four decades. In the early 2000s, the advent of *Wolbachia* transfection techniques allowed for broader application of IIT, both alone and combined with irradiation (Table 2).

Pilot IIT trials were generally designed to evaluate incompatible males’ mating competitiveness, dispersal, and survival, together with their capacity to induce a reduction in the egg fertility in the target area [288,289,290,291], while larger-scale trials were necessary to highlight the induction of a significant decrease in the number of biting females, providing evidence regarding the potential of IIT to reduce the epidemiological risks [161,220,292,293,294].

The earliest field work within the transfection-based renaissance occurred in French Polynesia and involved a population of *Ae. polynesiensis* introgressed with *Wolbachia* from *Ae. riversi* [291]. Subsequently, early tests of localized IIT suppression (i.e., of individual home properties) occurred with *Ae. albopictus* in suburban areas in the USA [290] through the release of males infected with *Wolbachia w*Pip from *Cx. pipiens* [89,295]. After that, several further pilot or large-scale IIT trials targeted the latter species (Table 2). In some trials involving invasive transinfected strains and a lack of sufficiently accurate sexing protocols, IIT has been combined with the irradiation of the mosquitoes to be released (Table 2). As an example, open field IIT trials conducted in China in July 2016 to suppress *Ae. albopictus* [161] made use of a transinfected population carrying a triple *Wolbachia* infection, established by adding *Wolbachia w*Pip to the native *Wolbachia* strains. Prior to large-scale releases for this trial, mosquitoes were irradiated to avoid the risk that the triple infection could spread locally (Section 2.1 and Section 2.2). The experiments led to the near elimination of the local population of *Ae. albopictus*, however, the added value of combining IIT and SIT in terms of sustainability is debated [296].

*Ae. aegypti* has also been a target of IIT trials (Table 2), even if this species, being uninfected by *Wolbachia* in nature, is more sensitive to risks of undesired population replacement (see Section 2.2). In Singapore, the National Environmental Agency (NEA) has coordinated an IIT program since 2016 using male *Ae. aegypti* mosquitoes infected with the *Wolbachia w*AlbB strain. IIT releases led to strong reductions in the wild population of *Ae. aegypti* and a 77.28% reduction in dengue transmission across all release sites despite incomplete coverage [264,293]. Public acceptance has been strong, aiding implementation and scalability, especially as the released mosquitoes are non-biting [284]. However, challenges remain, including higher upfront costs due to the need for large-scale and continuous releases, as well as logistical demands like sex separation and irradiation of mosquitoes [297]. Other IIT trials against *Ae. aegypti* were successfully conducted in California [220], Florida [292], Puerto Rico [298], and Texas [299] (USA), in Queensland [300] (Australia), in the Caribbean [301], and, in combination with SIT, in Mexico [138] and Thailand [302], in all cases leading to a strong reduction of the number of biting females (Table 2). Within most recent IIT trials, the Debug program conducted by Verily (Alphabet’s life sciences arm) [303] in collaboration with other partners (Table 2) started experimenting with an automated mosquito-rearing and AI-based sexing system (see Section 2.1.1) that could contribute significantly to increasing the sustainability of IIT programs [220].

Indeed, despite perspectives of further implementation that are encouraged by the demonstrated effectiveness of the strategy, sustainability is the main issue of IIT because, as this strategy is self-limiting (see Section 2.1.1), IIT programs generally require a long-term business plan and collaboration between public and private bodies to support the investments that are necessary. This is why the exploitation of IIT on a large scale is generally accompanied by pesticide registration applications and marketing authorizations (see Section 2.3).

#### 2.5.2. Population Replacement Strategy

As shown in Table 2, the first population replacement releases took place in Cairns, Australia in 2010 targeting *Ae. aegypti* with the *w*Mel *Wolbachia* strain, resulting in the establishment of *w*Mel at high frequencies at two independent sites [304]. The *w*Mel strain has persisted at high frequencies in these locations for over a decade [261,305]. Releases of *Ae. aegypti* carrying *w*Mel for population replacement have since expanded across tropical Queensland, Australia [108,306] and to over 10 other countries within the World Mosquito Program (WMP; [307]) (Table 2). For instance, in Brazil, the implementation of *Wolbachia*-transinfected *Ae. aegypti* mosquitoes began in 2014 with their release and successful establishment in the cities of Rio de Janeiro and Niterói [308]. While *w*Mel is the most widely released strain, other strains including *w*MelPop [244] and *w*AlbB [141] have been released in some countries. To date, population replacement programs have not targeted other vector species. Although unidirectionally compatible *Wolbachia* strains have been developed in *Ae. albopictus* that would be suitable for population replacement [79,80,160], few studies have evaluated their effects on arbovirus transmission (Table 1).

Most published *Wolbachia* population replacement programs have been successful in achieving their aim of establishing *Wolbachia* transinfections in the target population [99,294,309]. While population replacement is often stable and self-sustaining once reaching high frequencies, releases in some locations have been more challenging, requiring supplemental releases to maintain the *Wolbachia* transinfection in the population [100,310,311].

Following the establishment of *Wolbachia* in natural populations, several studies have now demonstrated substantial impacts of releases on dengue cases [108,114,141,196,306,312,313] and other arboviruses [196] through both experimental trials and operationalized releases. While estimating the true impact of *Wolbachia* releases on arbovirus transmission is challenging due to the dynamic nature of both human and mosquito populations [314], estimates of reductions in dengue cases are typically above 40% and as high as 90% or above in trial sites where *Wolbachia* transinfections have reached high frequencies (Table 2).

Efficacy also depends on the frequency of *Wolbachia* in the population, with locations where *Wolbachia* frequencies are low showing weaker impacts of *Wolbachia* releases on dengue cases [101,315]. Indeed, while most of the introgression programs were successful, the large *Wolbachia* (*w*Mel strain) release program carried out in 2017 in Rio de Janeiro, Brazil [101] exemplifies the challenges associated with implementing release programs. On average, 32% of mosquitoes collected from the release zones between 1 and 29 months after the initial release tested positive for *w*Mel. Reduced *w*Mel introgression was observed in locations and seasonal periods with historically high cases of dengue and chikungunya, with the percentage of mosquitoes testing positive for *w*Mel decreasing to 25% during peak disease incidence months. The study cited seasonal temperature fluctuations as a possible reason for the incomplete introgression observed (see Section 2.7). Nevertheless, the releases were associated with a 38% reduction in dengue incidence and a 10% reduction in chikungunya incidence. More recently, a cluster-randomized controlled trial (EVITA Dengue; registered with ClinicalTrials.gov (NCT04514107)) was designed and conducted in Belo Horizonte to generate high-quality data on the effectiveness of releasing *Wolbachia*-infected *Ae. aegypti* mosquitoes in reducing the incidence of arboviral infections [316]. The insights gained from this study will inform the global implementation of this method and contribute to strengthening the evidence base for integrated arboviral disease prevention strategies.

The results from these studies indicate the viability of such *Wolbachia*-based interventions, however, the differing efficacies reported suggest that further work is required to evaluate the cost-effectiveness of these programs and understand factors that affect the ability of *Wolbachia* to establish in disparate real-world settings. If *Wolbachia* transinfections can be maintained in populations in the long term, replacement releases will likely provide ongoing protection against dengue and other arboviral diseases, with studies monitoring populations across multiple years demonstrating sustained impacts on dengue cases [294,311,313]. Furthermore, laboratory studies show that *Wolbachia*-infected mosquitoes collected from field release sites have maintained their ability to block dengue transmission [110,142,317].

**Table 2:** Open field vector control trials and operational programs based on the Incompatible Insect Technique (IIT) and on the Population Replacement Strategy (PRS).

Program Name (If Any)/Region	Open Field Activities ^a^	Target Species	Involved *Wolbachia* Infection (Name of the Transinfected Line, If Any)	Control Strategy	Level of the Intervention ^b^	Target Area ^c^	Measured Effect ^d^	Partners and Supporters ^e^
-	2012	*Ae. polynesiensis*	*w*Riversi (CP [163])	IIT (at about 0.6:1 release ratio)	Pilot trial [291]	French Polynesia	Significant reduction of adult females	Public Bodies Institut Louis Malardé (French Polynesia); Government of French Polynesia; University of Kentucky (Lexington, KY, USA); National Institutes of Health (USA) Private Bodies Bill and Melinda Gates Foundation (Seattle, WA, USA)
-	2014	*Ae. albopictus*	*w*Pip (AR*w*P_US_; [295])	IIT (release ratio not available)	Pilot trial [290]	Lexington (KY, USA)	Significant reduction of egg hatching rate; Significant reduction of adult females	Public Bodies University of Kentucky (KY, USA); Kentucky Cabinet for Economic Development; National Institutes of Health (KY, USA) Private Bodies MosquitoMate, Inc. (Lexington, KY, USA)
-	2015–2018	*Ae. albopictus*	*w*AlbA + *w*AlbB + *w*Pip (HC line [161])	IIT-SIT combined (at 10–50:1 release ratio)	Large-scale trial [161]	Guangzhou (China)	>94% reduction of the wild population	Public Bodies *China*: Sun Yat-sen University in Guangzhou; Hunan Normal University; Guangzhou Center for Disease Control and Prevention; Center for Applied Mathematics, College of Mathematics and Information Sciences, Guangzhou University; School of Medicine, Hunan Normal University, Changsha; Nanjing Agricultural University; Guangdong Provincial Center for Disease Control and Prevention; National Natural Science Foundation of China; Chinese Center for Disease Control and Prevention, Beijing; Natural Science Foundation of Hunan Province, Hunan CDC, Hunan Educational Committee, Hunan Province *Other countries*: Michigan State University (MI, USA); IAEA (Joint FAO/IAEA, Programme of Nuclear Techniques in Food and Agriculture, Vienna International Centre, Austria); University of Melbourne (Australia) Private Bodies Guangzhou Wolbaki Biotech Co. (Guangzhou, China)
-	2016	*Ae. aegypti*	*w*AlbA + *w*AlbB (ThAB line [153])	IIT-SIT combined (release ratio not available)	Pilot trial [302]	Plaeng Yao District (Thailand)	85% reduction of egg hatch rate; 97% reduction of adult females	Public Bodies *Thailand*: Mahidol University Hua Sam Rong Administrative Authority, Plaeng Yao District Health Office, Plaeng Yao Hospital, Nong Satit School *Other countries*: International Development Research Centre (IDRC, Canada); International Atomic Energy Agency (IAEA, Austria)
-	2019	*Ae. aegypti*	*w*AlbB (introgression from WB2 line [68])	IIT-SIT combined (at 10:1 estimated release ratio) within an IVM plan	Large-scale trial	Merida (Mexico)	76–0–88% reduction of egg hatch rate (depending on the phase of the experiment); 55–61–75% reduction of biting females (depending on the phase of the experiment)	Public Bodies *Mexico*: Ministry of Health (MoH); Collaborative Unit for Entomological Bioassays (UCBE) and Laboratory of Biological Control (LCB) of Autonomous University of Yucatan (UADY); Fondo Mixto Consejo Nacional de Ciencia y Tecnología; Gobierno del Estado de Yucatán *Other countries*: University of Michigan (MI, USA); U.S. Agency for International Development (USAID)
*Wolbachia* Singapore	2016–present	*Ae. aegypti*	*w*AlbB	IIT and IIT/SIT combination (release ratio not available)	Operational program [264,293,297]	Singapore	>90% reduction of the wild population after 12 months of sustained intervention; 56–88% reduction of dengue incidence [264,293]; 61% reduction of dengue incidence after 12 months of sustained intervention [297]	Public Bodies *Singapore*: National Environment Agency (NEA); Singapore Ministry of Finance, Ministry of Sustainability, and the National Environment Agency; Singapore National Robotics Program Private Bodies Verily Life Sciences LLC (South San Francisco, CA, USA); Orinno Technology Pte. Ltd. (Singapore)
Debug/Debug Fresno	2017–2018	*Ae. aegypti*	*w*AlbB (WB1 [68])	IIT (release ratio not available)	Large-scale trials [220]	Fresno (CA, USA)	95% reduction of the wild population	Public Bodies University of Kentucky (KY, USA); Consolidated Mosquito Abatement District (CMAD) (CA, USA) Private Bodies Verily Life Sciences LLC (South San Francisco, CA, USA); MosquitoMate (Lexington, KY, USA)
	2018	*Ae. aegypti*	*w*AlbB (WB1 [68])	IIT (release ratio not available)	Large-scale trial [292]	Miami (FL, USA)	Significant reduction of egg hatching rate; 78% reduction of adult females	Public Bodies Florida Department of Health (FL, USA); Mosquito Control Division, Department of Solid Waste Management, Miami-Dade County (FL, USA) Private Bodies MosquitoMate (Lexington, KY, USA), Clarke Mosquito Control Services (St. Charles, IL, USA)
Innisfail Mozzie Program/Debug Innisfail	2018	*Ae. aegypti*	*w*AlbB (*w*AlbB2-F4 line)	IIT (5–10:1 release ratio)	Large-scale trial [300,318]	Innisfail (Queensland, Australia)	Significant reduction of larval productivity; >80% reduction of adult females	Public Bodies *Australia*: University of Queensland; CSIRO; James Cook University; QIMR Berghofer Medical Research Institute; Australian National Health and Medical Research Council *Other countries*: Michigan State University (MI, USA) Private Bodies Verily Life Sciences LLC (South San Francisco, CA, USA)
AR*w*P	2018–2019	*Ae. albopictus*	*w*Pip (AR*w*P line [89])	IIT (0.7–1.1:1 ratio releases)	Pilot trials [288,289]	Rome (Italy)	15–40% reduction of the egg hatch rate (depending on the year)	Public Bodies National Italian Agency for New Technologies, Energy, and Sustainable Economic Development (ENEA, Italy); Università degli Studi di Roma “La Sapienza” (Italy) Private Bodies BiovecBlok s.r.l. (Camerino, Italy, 2019–2024)
	2019	*Ae. aegypti*	*w*AlbB (WB1 line)	IIT (release ratio not available)	Large-scale trial [299]	Houston (TX, USA)	94% reduction of *Ae. aegypti* females; Significant increase of *Ae. albopictus* adults	Public Bodies University of Texas Medical Branch (TX, USA); Mosquito and Vector Control Division of Harris County Public Health (TX, USA); Texas Department of State Health Services (TX, USA) Private Bodies MosquitoMate, Inc. (Lexington, KY, USA)
	2020–2021	*Ae. albopictus*	*w*AlbA + *w*AlbB + *w*Pip (HC line)	IIT (at 1–7:1 release ratio)	Large-scale trial [80]	Changsha (China)	97–85% reduction of egg hatch rate (respectively, after once-, or twice-per-week releases); 94% reduction of mosquito biting	Public Bodies *China*: Hunan Normal University, Central South University; Sun Yat-sen University; Hunan Provincial Center for Disease Control and Prevention; Guangzhou Center for Disease Control and Prevention; Hunan Academy of Agricultural Sciences; National Natural Science Foundation of China; Natural Science Foundation of Hunan Province; Hunan CDC; Hunan Educational Committee *Other countries*: Michigan State University, (MI, USA) Private Bodies Guangzhou Wolbaki Biotech Co. (Guangzhou, China)
Communities Organized to Prevent Arboviruses (COPA) *Wolbachia* Project	2020–present	*Ae. aegypti*	*w*AlbB	IIT (release ratio not available)	Large-scale trial [298,301]	Ponce (Puerto Rico)	49% reduction of wild population [298]	Public Bodies *Puerto Rico*: Ponce Health Sciences University (Puerto Rico); Puerto Rico Vector Control Unit (Puerto Rico) *Other countries*: US Centers for Disease Control (GA, USA) Private Bodies Verily Life Sciences LLC (South San Francisco, CA, USA)
BugOut *Wolbachia*	2022–present	*Ae. aegypti*	*w*AlbB	IIT (release ratio not available)	Large-scale trial [319,320]	Virgin Gorda (British Virgin Islands)	Open field releases since 2022, data still not available	Public Bodies Government of Virgin Islands; Ministry of Health and Social Development Private Bodies Verily Life Sciences LLC (South San Francisco, CA, USA); GreenVI (Tortola, BVI)
World Mosquito Program—Australia/Dengue Safe Project Ingham/Dengue Out Program	2011–present	*Ae. aegypti*	*w*Mel	PRS	Operational program [321]	Cairns, Cassowary Coast, Douglas Shire, Charters Towers, Townsville (Queensland, Australia)	With a few local and often only momentary exceptions, mean *Wolbachia* (*w*Mel) frequency stably above 80–90% in treated areas; 96% reduction in dengue incidence in *Wolbachia*-treated populations [85,87,108,109,110,111,112,113,114,115,116,117,118,119,120,121,122,123,124,125,126]; Locally acquired dengue cases decreased to zero [322]	Public Bodies *Australia:* Monash University; Queensland Health Government; Queensland Government; Townsville Hospital and Health Service (Townsville HHS); Hinchinbrook Shire Council (HSC); Tropical Public Health Service; Northern Peninsula Area Regional Council; National Health and Medical Research Council of Australia; College of Public Health, Medical and Veterinary Sciences, James Cook University; Bio21 Institute, University of Melbourne *Other countries*: School of Public Health, University of California, (CA, USA); London School of Hygiene and Tropical Medicine (London, UK) Private Bodies WMP (Melbourne, Victoria, Australia); Bill & Melinda Gates Foundation (Seattle, WA, USA); Wellcome Trust (London, UK); Gillespie Family Foundation (New York, NY, USA); Foundation for the National Institutes of Health (Bethesda, MD, USA)
World Mosquito Program—Oceania	2018–present	*Ae. aegypti*	*w*Mel/Fij-*w*Mel, Van-*w*Mel, and Kir-*w*Mel	PRS	Operational programs [99,323]	Pacific islands of Oceania (Fiji, Kiribati, Vanuatu, New Caledonia)	Fiji: >80% *w*Mel prevalence in trapped *Ae. aegypti* in five of six reporting areas [99,324] Kiribati: intermediate *w*Mel prevalence (Eastern Site: 14.3–31.8%, Western Site: 50–100%) [99,320] Vanuatu: *w*Mel established in ten of the twelve reporting areas, with five reporting areas having >95% of *Ae*. *aegypti* infected with *w*Mel [99,325] New Caledonia: in Nouméa, *Aedes aegypti* individuals carrying *Wolbachia* reached 89%; in Mont-Dore (extended districts), *Aedes aegypti* individuals carrying *Wolbachia* reached 70%; in Dumbéa, *Aedes aegypti* individuals carrying *Wolbachia* reached 85% [323]	*Fiji* [324] Public Bodies Ministry for Health and Medical Services (Government of Fiji); Australian Government, Department of Foreign Affairs and Trade (Australia); USAID (USA); New Zealand Foreign Affairs and Trade, Aid Program (New Zealand) Private Bodies WMP (Melbourne, Victoria, Australia); Live and Learn Environmental Education (Melbourne, Victoria, Australia); Rotary Foundation (Evanston, IL, USA) *Kiribati* [326] Public Bodies Ministry for Health and Medical Services (Government of Kiribati); Australian Government, Department of Foreign Affairs and Trade (Australia) Private Bodies WMP (Melbourne, Victoria, Australia) *Vanuatu* [325] Public Bodies Ministry of Health (Government of Vanuatu); Australian Government, Department of Foreign Affairs and Trade (Australia) Private Bodies WMP (Melbourne, Victoria, Australia); Vanuatu Red Cross (Port Vila, Vanuatu) *New Caledonia* [323] Public Bodies Insitute Pasteur de Nouvelle Calédonie; Government de la Novelle-Calédonie; Ville de Nouméa; Ville du Mont-Dore; Ville de Dumbéa; Province Sud; Haut-Commissariat de la République en Nouvelle-Calédonie (France); Fonds Pacifique-Republique Française (France); Health Security Initiative 2017–2022 Private Bodies WMP (Melbourne, Victoria, Australia)
World Mosquito Program—Brazil	2015–present	*Ae. aegypti*	*w*Mel	PRS	Operational program [196,316,327,328]	Rio de Janeiro, Niterói, Belo Horizonte, Campo Grande, Petrolina (Brazil)	Rio de Janeiro: 25–32% introgression of *Wolbachia w*Mel in the wild population, 38% reduction in dengue incidence, 10% reduction in chikungunya incidence [101]; Niterói: 40–80% introgression of *Wolbachia w*Mel in the wild population, 69.4% reduction in dengue incidence, 56.3% reduction in chikungunya incidence, 37% reduction in Zika incidence [196]; Belo Horizonte, Campo Grande, and Petrolina: ongoing studies, data still unavailable	Public Bodies *Brazil*: Oswaldo Cruz Foundation (Fiocruz, Brazil), Ministry of Health of Brazil; various Community Reference Groups (see [328]). *Other countries*: Monash University (Melbourne, Australia); European Research Council Private Bodies WMP (Melbourne, Victoria, Australia); Bill & Melinda Gates Foundation (Seattle, WA, USA)
World Mosquito Program—Colombia	2017–present	*Ae. aegypti*	*w*Mel (*w*Mel-COL/*w*Mel-COL2) [100])	PRS	Operational program [100,329]	Bello, Medellin, Itagui	Bello: 81.1–96.6% introgression of *Wolbachia w*Mel in the wild population [100]; About 95% reduction of dengue incidence [312]; Medellín: extremely variable results regarding the percentage of *Wolbachia* introgression, ranging from 18.4–98.1%, depending on the area and on the period [100]; 9.5–33.2% introgression of *Wolbachia w*Mel in the wild population [330]; About 95% reduction of dengue incidence [312]; Itagui: 63.6–92.3% introgression of *Wolbachia w*Mel in the wild population [100]; About 97% reduction of dengue incidence [312]	Public Bodies *Colombia*: Universidad de Antioquia; Secretaría de Salud, Medellín *Other countries*: U.S. Agency for International Development (USAID, USA); UK Department for International Development (UK) Private Bodies WMP (Melbourne, Victoria, Australia); Bill & Melinda Gates Foundation (Seattle, WA, USA); Wellcome Trust (London, UK)
World Mosquito Program—Central America	2019–present	*Ae. aegypti*		PRS	Large-scale trials [331,332,333]	Central America (Mexico, Honduras, El Salvador)	Mexico: open field releases since 2019, data still not available Honduras: open field releases since 2023, data still not available El Salvador: open field releases since 2024, data still not available	*Mexico* Public Bodies Secretaria de Salud Gobierno de Baja California Sur (Mexico) Private Bodies WMP (Melbourne, Victoria, Australia), International Community Foundation (ICF, National City, CA, USA); Wellcome Trust (London, UK); Alumbra Innovations Foundation (Bentonville, AR, USA) *Honduras* Public Bodies Universidad National Autonoma de Honduras; Secretaria de Salud, Gobierno de Honduras Private Bodies WMP (Melbourne, Victoria, Australia); Medecins sans Frontieres (Geneva, Swiss ) *El Salvador* [333] Public Bodies Gobierno de El Salvador, Ministerio del Salud; PRVCU International (Unidad de Control De Vectores de Puerto Rico, Puerto Rico) Private Bodies WMP (Melbourne, Victoria, Australia)
World Mosquito Program—Vietnam	2013–present	*Ae. aegypti*	*w*MelPop *w*Mel	PRS	Operational program [283]	Vietnam	Failure of *w*MelPop *Wolbachia* infection establishment [244]; heterogeneity in *w*Mel *Wolbachia* infection prevalence [242]	Public Bodies Institute Pasteur Vietnam; Ministry of Health of Vietnam, Action on Poverty; National Institute of Hygiene and Epidemiology of Vietnam Private Bodies WMP (Melbourne, Victoria, Australia)
Applying *Wolbachia* to Eliminate Dengue (AWED)/World Mosquito Program—Indonesia	2017–2020	*Ae. aegypti*	*w*Mel	PRS	Large-scale trials [294]	Yogyakarta, Indonesia	95.8% *Wolbachia* introgression in intervention clusters; 77.1% reduction of dengue cases; 86.2% reduction of hospitalizations [294]	Public Bodies Universitas Gadjah Mada, Indonesia Private Bodies WMP (Melbourne, Victoria, Australia), Tahija Foundation (Jakarta, Java, Indonesia)
World Mosquito Program—Laos and Sri Lanka	2021–present	*Ae. aegypti*	*w*Mel	PRS	Operational program [334,335]	Laos, Sri Lanka	Laos: open field releases since 2022, data still not available Sri Lanka: open field releases since 2021, data still not available	*Laos* [335] Public Bodies Ministry of Health of Laos Private Bodies WMP (Melbourne, Victoria, Australia), Save the Children (London, UK) *Sri Lanka* [334] Public Bodies National Dengue Control Unit of Sri Lanka Private Bodies WMP (Melbourne, Victoria, Australia), Australian Aid (Camberra, ACT, Australia)
*Wolbachia* Malaysia	2017–present	*Ae. aegypti*	*w*AlbB (*w*AlbB.MC line)	PRS	Operational program [141,142,311,336,337]	Malaysia	*w*AlbB frequency in the wild population at 98% in one year in release sites and reduction in dengue incidence higher than 40.3% [141]; *w*AlbB frequency in the wild population higher than 80% in release sites [336]; average reduction in dengue fever of 62.4% [311]; 37.69% reduction of dengue incidence in adjacent non-intervention areas [313]	Public Bodies *Malaysia*: Ministry of Health Malaysia; Institute for Medical Research; Health Department of Federal Territory of Kuala Lumpur & Putrajaya *Other countries*: 3MRC-University of Glasgow Centre for Virus Research (UK); University of Melbourne (Australia); Telethon Kids Institute, Perth Children’s Hospital, (Australia); Curtin University (Australia); Australian National Health and Medical Research Council Private Bodies Wellcome Trust (London, UK)

IIT = Incompatible Insect Technique; SIT = Sterile Insect Technique; PRS = Population Replacement Strategy. ^a^ Information is grouped by strategy with IIT trials presented first followed by Population Replacement trials; trials are ordered chronologically based on the start of open field releases; ^b^ Pilot Trials: studies that are conducted on a small scale to analyze certain specific biological parameters and assess preliminarily the effectiveness of a control strategy and that are addressed to the setup of larger-scale trials; Large-Scale Trials: experimentations that are conducted on a larger scale and for a longer period to test effectiveness and feasibility of the control strategy under operational conditions; Operational Programs: control programs planned in collaboration with the local institutions to reach a defined objective in the long term. ^c^ Main city, or province, and country are generally indicated while the exact locations can be found in the cited reference. ^d^ Reported data represent the best achieved result before the interruption of the releases (in the case of IIT) or at the end of the program. ^e^ Data regarding partners and supporters refer to both the open field trials and the studies to ascertain their results.

### 2.6. Cost-Effectiveness of Mosquito Control with a Specific Focus on *Wolbachia*-Based Disease Control Strategies

A limited number of studies evaluating the cost-effectiveness of mosquito control methods or their sustainability are available [280,338,339,340,341], mainly due to the difficulty in measuring non-market values, including the preservation of biodiversity, ecosystems, and cultures, by choosing the opportune indexes in each specific scenario [342]. However, these principles should be taken into account especially by large-scale mosquito control programs.

As already mentioned in Section 2.1, in the evaluation of a vector control strategy, two requirements should be met: (i) demonstration of safety, quality, and entomological efficacy of the proposed method; (ii) evidence that it reduces disease in the target vector population [343]. Conventional strategies for prevention and control of arboviral diseases primarily involve preventing mosquito bites, implementing vector control measures, and engaging communities in environmental management initiatives [344]. In evaluating the cost-effectiveness of these strategies, existing literature has mainly focused on examining the effects of vaccination [345,346] or vector control programs [347,348,349,350] singularly. Shepard et al. [345] demonstrated that the cost per disability-adjusted life year (DALY) saved by a pediatric vaccine would be USD 50, making the potential vaccine highly cost-effective. Meanwhile, Suaya et al. [349] found that annual targeted larvicidal campaigns against *Ae. aegypti* would grossly cost USD 567,800 per year, or USD 0.20 per person covered, resulting in USD 313/DALY gained from the public perspective and USD 37/DALY gained from the societal perspective. In Brazil, Pepin et al. [350] reported that the implementation of a novel mosquito surveillance and control system prevented 27,191 cases of dengue fever and saved an average of USD 227 (median USD 58) per case prevented, which saved approximately USD 364,517 in direct costs (healthcare and vector control) and USD 7,138,940 in lost wages (societal effect) annually. However, in practice, control strategies are often implemented in an integrated manner [351,352]. As such, the costs and benefits of these strategies need to be considered as a combined approach. Knerner et al. [353] used a dynamic transmission model to show that a combination of vaccination, adulticide, larvicide, and public engagement would result in 208 DALYs lost per million population. For reference, the individual strategies would result in 506, 657, 942, and 814 DALYs lost per million population, respectively, when implemented as single vector control interventions or vaccination strategies. While the cost-effectiveness of integrated vector control strategies varies based on a multitude of factors (e.g., local context, disease burden, and implementation practices), the above results validate integrated vector control strategies as a valuable investment in public health.

As evidenced in Section 2.5, *Wolbachia*-based control programs have had varying entomological efficacies. This poses an additional challenge when evaluating cost-effectiveness, as the entomological efficacy may not directly translate to epidemiological outcomes. Consequently, this evaluation has been mainly conducted through simulation and modeling studies [294,337,353,354,355,356,357,358,359]. Brady et al. [355] predicted that a program of *Ae. aegypti* population replacement would have a gross cost-effectiveness below USD 1500 per DALY averted when deployed in high-density urban areas such as that of Yogyakarta, Indonesia. In Brazil, the economic impact of implementing a similar program was estimated to yield a cost difference of USD 538,233.68 and avert 5.56 DALYs with net monetary benefits ranging from USD 110.72 to USD 1399.19 per inhabitant [354]. In Singapore, Soh et al. [356] estimated that an IIT program would cost an estimated USD 50,453–100,907 per DALY averted and would lead to an estimated USD 329.40 million saved in economic costs from 2010 to 2020 under 40% intervention efficacy and an assumed steady-state running cost of a program at the national level. The large discrepancy in estimated cost-effectiveness arises due to the different implementations of the *Wolbachia*-based control programs in these countries. In Indonesia and Brazil, cost-effectiveness was estimated for PRS, while in Singapore, estimates were made for IIT for which field trials have demonstrated both entomological and epidemiological efficacy [264,293,297]. The cost-effectiveness of control programs is also highly contextual, relying fundamentally on healthcare and economic costs incurred from arboviral diseases, which can vary significantly across low-, middle-, and high-income nations. This consequentially affects the cost savings which can result from preventing cases of arboviral infections under *Wolbachia*-based control programs.

Costs can also significantly vary between PRS and IIT programs due to operational reasons. While PRS requires significant initial investments for extensive mosquito releases to establish *Wolbachia* within the population, suppression programs require regular, long-term, large releases or interventions to maintain low mosquito density, generally requiring precise sexing methods that can additionally affect costs and overall sustainability [355] (Table A1). For both strategies, long-run costs may also be incurred from the need for close entomological monitoring to ensure stable introgression/suppression for the respective programs. Crucially, cost-effectiveness also depends on the estimated efficacy/effectiveness of the assessed program.

### 2.7. Effects of Climate Change on Mosquito-Borne Diseases and on *Wolbachia*-Based Control Strategies

Climate change is expected to alter the geographic distribution and abundance of many species, including arthropod vectors of diseases [16,360]. Arthropods are ectothermic, and climate change, especially warming temperatures, will affect their reproduction, survival, geographic distribution, relative abundance, and ability to transmit pathogens [17]. For example, the viability of vectors responds strongly and non-linearly to temperature and other climate variables, and therefore to climate change [361,362], as shown by Jia et al. [363], Pasquali et al. [364], Gutierrez et al. [365], and others using mechanistic and correlative approaches [366,367]. Further, the management of complex vector-borne diseases is complicated by interactions in the system [368]. In addition, physiological adaptation to temperature in mosquito vectors is an important factor mostly overlooked when exploring the effects of climate change on mosquito-borne diseases [369]. Given this complexity, the capacity to model explicitly the dynamics and interactions of the different components of eco-epidemiological systems is crucial to assessing and managing them especially when pursuing the efficacy, safety, and sustainability of *Wolbachia* as a disease vector control measure.

The study of the transmission of vector-borne diseases must consider the panoply of biological, ecological, socioeconomic, demographic, and human-caused factors, with climate variables being major driving factors determining the potential risk and burden of both vectors and pathogens at extant local and regional levels and their potential range expansion or contraction under climate change [370,371]. Climate warming and global transport are expected to increase the risks of mosquito-borne diseases, especially arboviruses, in novel areas by increasing the geographic range of the vectors [11,12,13,14,15,16,17,361,372,373,374] and the associated epidemic potential [375].

The general effects of temperature on poikilotherm systems are well known but must be documented for the vector, *Wolbachia* strains, and the diseases in the context of extant weather, including climate change. For example, within the host, *Wolbachia* density is affected by temperature [98,106,149,376,377,378,379,380], especially during the mosquito larval stage, and this can reduce both the CI induced by *Wolbachia* and its maternal transmission (Table A1) [103,106,149]. Partial reduction of *Wolbachia* infections in mosquito populations may reduce their ability to block arbovirus transmission [103]. In turn, *Wolbachia* can affect temperature tolerance of infected mosquito vectors [379,380]. Temperature may also interact with other factors and a prolonged duration of the egg stage (due to natural diapause or storage in artificial conditions) can adversely impact the *Wolbachia*–host interactions (Section 1.4 and Section 2.1 and Table A1).

Host fitness costs of *Wolbachia* infection and the vulnerability of some strains of *Wolbachia* to environmental conditions may help to explain seasonal fluctuations in infection frequencies and establishment success in some environments [141,196,242]. Environmental constraints on *Wolbachia* require assessments of multiple strains to determine which is the best fit to specific environmental conditions. Further, the ability of a *Wolbachia* strain to establish and increase in frequency may shift as temperatures increase and/or rainfall patterns shift with climate change. However, estimates of regional suitability will require a better understanding of the ecological and climatic factors that drive *Wolbachia* dynamics in mosquitoes which are currently poorly understood [378].

## 3. Discussion

### 3.1. Perspectives of Implementation of *Wolbachia*-Based Control Strategies and Possible Issues

A road map for neglected tropical diseases supports a cross-sectoral strategy that encompasses the One Health approach [381,382] and the measures articulated in the Global Vector Control Response (GVCR) 2017–2030 by the WHO [281]. The WHO also launched in 2022 a more specific Global Arbovirus Initiative (GLAI), with the aim to build an integrated strategic plan to tackle emerging and re-emerging arboviruses with epidemic and pandemic potential, focusing on risk monitoring and detection, pandemic prevention, and quick response [383,384]. In this context, innovative vector control methods must demonstrate clear entomological and epidemiological efficacy and should prioritize safety and sustainability (see Section 1.3 and Section 2.6) to be recommended.

Based on the evidence of efficacy against arboviral diseases (Section 2.5 and Section 2.6), the use of *Wolbachia*-based PRS to reduce the vector competence of mosquitoes has already received a favorable recommendation from the WHO Vector Control Advisory Group [385]. Regarding IIT, the data supporting efficacy against arbovirus transmission are gradually increasing (Section 2.5.2). However, implementation at a large scale remains challenging, mainly because operational demands are more resource-intensive compared to PRS (Section 2.5.1). Despite this, an increase in IIT-based operational programs is expected because this approach offers some long-term advantages over the introgression method, such as avoiding the potential for arboviruses to develop resistance to *Wolbachia* [258] (Section 2.3) and concerns about *Wolbachia* stability under climate change [125] (Section 2.6). Furthermore, new technologies able to increase the sustainability of IIT could support its deployment in further areas (see Section 3.3). Choosing PRS or IIT can also be influenced by the target area, with the tropics possibly more indicated for PRS, because of the endemicity of several arboviruses, and temperate areas more suitable for IIT as the risks of epidemics are restricted only to a short period of the year and population replacement can be viewed as not strictly necessary. However, climate change and a further spread of arboviruses and vectors can make any scheme too simplistic. To confirm this, while a majority of projects have until now focused on mosquito populations in tropical and subtropical areas where arbovirus epidemics are endemic, there is a growing interest in the use of *Wolbachia* approaches in temperate areas [386,387], in part due to the increasing frequency of arbovirus epidemics in areas previously not thought to be at risk.

Despite the reassurances coming from risk assessment studies (see Section 2.2), further implementation of both *Wolbachia* approaches should be accompanied by vigilance regarding any unforeseen negative impact in the field. This includes having robust emergency plans and alternative techniques in place to reverse the primary methodology if necessary [388].

Despite the potential of *Wolbachia* as a control tool, PRS and IIT have been used to control only a limited number of mosquito vectors (Table 2) due to characteristics of the species or to their unsuitability for *Wolbachia* infection (Section 2.1.1; Table 1; Figure 3). This latter issue, for instance, currently impedes the use of *Wolbachia* to combat the burden of malaria. However, the transinfection of *Anopheles stephensi* has been proven feasible (Table 1), and in both this species and *An. gambiae*, generally not infected by *Wolbachia* in nature, this endosymbiont seems to inhibit *Plasmodium* infection [389]. Even if the relevance of a few records of the presence of *Wolbachia* in natural *An. gambiae* population is debated [390], establishing a stable and exploitable *Wolbachia* infection in this key vector cannot yet be considered absolutely unfeasible.

Evaluating the cost-effectiveness of traditional vector control programs and *Wolbachia* interventions is critical for optimizing resources and maximizing the impact of arboviral disease control efforts. Compared to other traditional control strategies (Section 1.2), *Wolbachia* approaches involve significant initial investments for releasing infected mosquitoes, yet they promise substantial long-term benefits by reducing vector populations and disease transmission. Integrated evaluations considering both traditional and innovative control strategies and their combined use with the support of landscape ecology and urban science [391] can provide a more comprehensive understanding of their economic viability and effectiveness. Such assessments are essential for informing policy decisions, ensuring sustainable public health interventions, and ultimately reducing the burden of dengue and other mosquito-borne diseases.

### 3.2. The Importance of Involving Public and Private Partners and Communities

As shown in Table 2, large-scale operational programs have involved several partners and sponsors, including both public and private bodies. The support by citizens, gained by opportune informative campaigns and, in some cases, through direct involvement, has also been fundamental for achieving acceptance and support by all the community (Section 2.4). A coordination of the efforts is advocated by the application of the One Health principles to arboviral disease control [381] because addressing zoonotic public health threats, environmental issues, and neglected tropical diseases as a whole is considered a key for a better understanding of disease dynamics. This approach is expected to facilitate the development of comprehensive and sustainable strategies for disease prevention, control, and eradication able to bring together and coordinate the relevant stakeholders and sectors involved in this field [392]. The success of several PRS and IIT trials demonstrates that coordinating the efforts of all the involved stakeholders is fundamental to program success. Future implementation of *Wolbachia*-based control strategies will have to take into account this lesson.

### 3.3. Development of Models to Enhance Cost-Effectiveness of *Wolbachia*-Based Control Strategies

The eco-epidemiological complexity of vector-borne disease systems has historically hampered successful field implementation of vector control strategies and efforts [393,394,395]. Understanding and managing the spread of mosquito-borne diseases necessitates sophisticated modeling approaches that integrate various biological and environmental data. Mathematical models are a powerful way to explore optimization of *Wolbachia*-based strategies for vector-borne disease control because, coupled with field data, they can support the identification of the most effective and most logistically feasible control strategy prior to open field deployment [396]. However, while identifying critical components of the system, many mathematical models make simplifying assumptions about the potential limiting effects of the environmental drivers (say temperature and rainfall patterns) and about demographic parameters. In contrast, holistic weather-driven population dynamics models can capture mosquito population behaviors, including growth rates, lifespan, and epidemiological interaction patterns [353]. Such models would serve as crucial inputs for mosquito-borne disease transmission models to simulate the spread of diseases within human populations and help optimize strategies for releases of *Wolbachia*-infected mosquitoes to improve efficiency and coverage and to contain costs. Specifically, in the case of IIT, modeling approaches can address key questions to enhance efficacy and guarantee sustainability of operational programs: (i) when to start and stop releases; (ii) where to release; (iii) how many incompatible males to release; (iv) with which frequency; (v) at which ratio compared to wild males; (vi) with which acceptable frequency of female contamination to avoid undesired population replacement; (vii) whether incompatible male ratio or frequency of the releases can be reduced at a certain point to save costs without significantly affecting control.

Recent studies examined these topics by leveraging specific population dynamics models and provided valuable insights for a cost-effective implementation of IIT [397,398]. Even in the absence of target population eradication, an optimal stopping point of incompatible male releases can be found where control is sufficiently effective, which can reduce economic burdens in policy implementation [398]. Nevertheless, depending on the level of residual presence of *Wolbachia*-infected females escaping sexing, significant adjustments to the release protocols can be necessary. Pagendam et al. [397] examined optimal implementation of IIT programs by using a simple Markov population process model and determined the best deployment strategies in terms of overflooding ratio over time and maximum level of acceptable female contamination to achieve vector suppression but preventing *w*AlbB establishment of the *Wolbachia*-transinfected line. Population replacement programs too can certainly benefit from modeling studies. Cardona-Salgado et al. [399] used operations research methods to analyze dengue transmission, based on a model for *Ae. aegypti* that accounts for the presence of wild and *Wolbachia*-carrying vector transmitters, and concluded that it was more reasonable to prioritize the minimization of intervention time interval over the reduction of the total number of human infections during the intervention period. Dye and Cain [400], encouraged by the *Wolbachia*-based control of mosquitoes carried out successfully in Cairns, Australia, in 2011 that reduced dengue transmission, built upon a model with spatial and temporal dependence of diffusion coefficients in a reaction–diffusion model for an *A. aegypti* invasion [401].

Simulations like this help predict the outcomes of different intervention parameters without the need for initial large-scale field trials and allow for the identification of optimal strategies, potential challenges, and economic implications before implementing the interventions in real-world scenarios; however, they also emphasize the need for field experiments to validate the models in localized areas before large-scale applications are attempted.

Among factors driving vector–disease–host interactions, weather effects play a key role and the capacity to mechanistically model the complexity of the weather-driven ecology of the *Wolbachia*–vector–disease–host interactions would enable separation and understanding of vector abundance and disease frequency dynamics under extant weather and climate change [402,403].

Physiologically based demographic modeling (PBDM) of *Ae. albopictus* and *Ae. aegypti* [365,404] has been developed using data available in the literature that captured biological processes of the species as driven by daily weather, enabling projecting of their prospective geographic distribution and relative abundance across Africa, the European Palearctic, and North America [365]. The results concur with mappings using correlative species distribution models based on species distribution records [405]. Further, the PBDM allows incorporating new biological findings required for local and regional assessment of population suppression techniques such as *Wolbachia* endosymbionts (Section 2.1), insights that would be time-consuming and costly to obtain experimentally [406]. PBDM captures the weather-driven biology of species, hence its predictions are independent of time and place, including climate change effects [366], and adding additional realism does not alter the basic structure of PBDMs.

### 3.4. Biotechnological Methods to Support Sustainability of *Wolbachia*-Based Vector Control

As reviewed in Section 2.1.1, referring to IIT, sexing is a key step that is necessary before incompatible male releases that has a significant impact on costs, due to the time, devices, and personnel needed for the handling procedures but also because of the reduced yield compared to the total amount of reared individuals that consume resources and space [210]. While a number of genetic sexing strains have already been developed (Section 2.1.1; Table A1), their use could be restricted to specific programs and to countries where the release of GMOs is consented. RNAi-based methods could be a valuable alternative for sexing because the genetic modification of the insect to be released is not required. Administering the bioactive dsRNA through female-specific yeast larvicides [218] or through encapsulation [407] could restrict the exploitation of this genetic tool to mass rearing facilities. Additionally, in the case of the development of effective dsRNAs able to kill all female larvae by oral delivery [218], their addition to ready-to-use units furnished with eggs and larval food could pave the way to a more easy and sustainable retail distribution of the incompatible males.

Additional improvements of IIT protocols may regard the enhancement of the methods for packaging and transporting incompatible males, aiming at maximizing survival and at preserving the male mating competitiveness [408], and the exploitation of releasing strategies where robotics could support area-wide programs in reaching locations not easily accessible to human operators [409]. AI-driven approaches could be exploited to develop the best release protocols fitted to specific conditions [410].

As long as cost-effectiveness is demonstrated, all these strategies could contribute significantly to further implementing IIT.

## 4. Conclusions

In summary, our review highlights the recent progress and challenges of mosquito release programs for arbovirus control. Interventions have progressed rapidly in the last decade from small-scale trials to operational interventions which are now demonstrating strong efficacy against arboviral disease. With the increasing operationalization and commercialization of *Wolbachia* releases (as an example, see [411]), these types of interventions are likely to become a key component of integrated vector management programs. Interventions based on genetic constructs also show promise but are yet to have been released in the field and face additional challenges from both a regulatory and public support perspective but may provide important alternatives in situations where *Wolbachia* releases fail.

The outcomes of field releases to date provide important lessons for future interventions. Prerelease monitoring remains essential for targeting and optimizing releases and public support is crucial for any long-term intervention. For replacement releases, there may be biological constraints on where *Wolbachia* can spread, potentially requiring supplementary releases or alternate strains. Replacement can be highly heterogeneous and ongoing monitoring can help to identify areas where gaps need to be filled. For suppression, quality control is especially important to ensure that released males are competitive and that the release of fertile females is avoided which might jeopardize future control.

While *Wolbachia* releases are generally regarded as safe, with growing evidence of efficacy against arboviruses, several open questions remain about the long-term efficacy and sustainability of mosquito releases. How long are replacement releases likely to be effective for and can they be rolled out efficiently at broad scales, particularly given the potential issues with releasing a homogenous strain into heterogeneous landscapes with diverse mosquito genotypes [255]? Are replacement releases suitable for every environment or are there constraints, and can this be overcome by using different strains? For suppression interventions, how long will suppression last once releases cease, and are there any broader impacts on the ecosystem beyond mosquito species occupying the same ecological niche?

## Data Availability

Not applicable.

## Appendix A

**Table A1:** Genetic control strategies: characteristics and practical issues.

Control Strategy ^a^	Main Characteristics	Major Pros	Major Cons	Acceptance and Potential Risks	Main Specific Costs ^b^	Stage of Deployment ^c^
SIT [177]	Aimed at population suppression; self-limiting control strategy ^d^	Lack of requirement for a special mosquito strain; in the case of species easily suitable for laboratory rearing, it is possible to directly rear and release the population from the target area	Not all species are suitable for SIT because full male sterility is generally associated with a reduction in their fitness ^e^; sexing is required to avoid the release of biting females [206]	Generally accepted and regulated; no evidence of risks related to mutations induced by irradiation	Costs for irradiation ^f^ or for directly managing the radiation source	Open field trials [412,413]
IIT [287]	Aimed at population suppression; self-limiting control strategy	Lack of requirement for any prerelease treatment or genetic modification, so no added fitness costs, beyond effects of *Wolbachia*, and mosquitoes can be reared as normal aside from sexing	Not all species are suitable for *Wolbachia* infection; sexing is required to avoid the release of biting females and the spread of the new *Wolbachia* infection; very accurate sexing is required in Uni-CI-based IIT	Not accepted and regulated globally; risks of unintended population replacement which could reduce efficacy of further interventions	Initial costs to produce a *Wolbachia*-transinfected line; costs for monitoring *Wolbachia* frequencies in the case of non-perfect sexing methods	Open field trials (see Table 2)
SIT/IIT combination [414]	Aimed at population suppression; self-limiting control strategy	Lower risks of unintended population replacement compared to IIT, where contaminant females should be sterile; lower level of irradiation required compared to SIT	See cons related to both SIT and IIT	See both SIT and IIT regarding acceptance and potential risks	See main specific costs for both SIT and IIT	Open field trials (see Table 2)
*Wolbachia*-based PRS [415]	Aimed at population modification; self-sustaining control strategy if a threshold frequency is maintained	Lack of requirement for any prerelease treatment or genetic modification, so no added fitness costs, beyond effects of *Wolbachia*, and mosquitoes can be reared as normal; sexing is not required	Possibility of *Wolbachia* depletion or reduction of its effectiveness due to strain-specific constraints associated with climate; initial temporary increase in the number of biting females	Not accepted and regulated globally; risks of irreversible changes to native population and unintended impacts on arbovirus transmission	Initial costs to produce a *Wolbachia*-transinfected line; costs for monitoring *Wolbachia* frequencies	Open field trials, area-wide operational programs (see Table 2)
Transformation of mosquito vectors with CI-inducing genes from *Wolbachia* [171]	Aimed at population suppression; self-limiting control strategy	Lack of requirement for any prerelease treatment	Sexing is required to avoid the release of biting females and the spread of CI-inducing genes from *Wolbachia*; risks of inbreeding depression [192]	Not accepted and regulated globally due to the involvement of GMOs	Initial costs to produce the transgenic line; costs for monitoring persistence of transgenes	Laboratory assays [171,172]
Dominant lethal-based suppression (RIDL) [416]	Aimed at population suppression; self-limiting control strategy	Lack of requirement for any sterilization treatment; late-acting mortality	Sexing is required; use of antibiotics is required to switch off lethal genes for rearing; fitness costs; risks of inbreeding depression	Not accepted and regulated globally due to the involvement of GMOs	Initial costs to produce the transgenic line; costs for tetracycline treatments	Open field trials [417,418]
Doublesex suppression drive [185]	Aimed at population suppression; self-limiting control strategy	Lack of requirement for any prerelease treatment; strain is self-sexing so sexing is not required; population suppression acts across multiple generations	Use of antibiotics is required to switch off lethal genes for rearing; fitness costs; risks of inbreeding depression	Not accepted and regulated globally due to the involvement of GMOs	Initial costs to produce the transgenic line; costs for tetracycline treatments	Open field trials [185]
Transgenic sex ratio distortion [419]	Aimed at population suppression; self-sustaining control strategy	Lack of requirement for any sterilization treatment; the transgene is passed to the progeny that amplifies the effect across generations	Risks of inbreeding depression	Not accepted and regulated globally due to the involvement of GMOs	Initial costs to produce the transgenic line;	Semi-field trials [420,421]
CRISPR-based precision-guided SIT (pgSIT) [182]	Aimed at population suppression; self-limiting control strategy	Lack of requirement for any sterilization treatment; RNA-guided dominant knockout of specific genes determining male sterility and female inviability	Males to be released must be obtained by crossing two distinct strains; fitness costs; risks of inbreeding depression	Not accepted and regulated globally due to the involvement of GMOs	Initial costs to produce two transgenic lines; costs for maintaining two distinct transgenic strains	Laboratory assays [183]
CRISPR-based gene drive system targeting female reproduction [186]	Aimed at population suppression; self-sustaining control strategy	Lack of requirement for any sterilization treatment; the mutation is passed to the progeny that amplifies the effect across generations	Risks of inbreeding depression	Not accepted and regulated globally due to the involvement of GMOs	Initial costs to produce the transgenic line	Laboratory assays [186]
CRISPR-based gene drive system targeting female vector competence [235]	Aimed at population modification; self-sustaining control strategy	Lack of requirement for any sterilization treatment; the mutation is passed to the progeny that amplifies the effect across generations	Risks of inbreeding depression	Not accepted and regulated globally due to the involvement of GMOs	Initial costs to produce the transgenic line	Laboratory assays [235]

CI = Cytoplasmic Incompatibility; Uni-CI = Unidirectional Cytoplasmic incompatibility; Bi-CI: Bidirectional Cytoplasmic Incompatibility; SIT = Sterile Insect Technique; IIT = Incompatible Insect Technique; GMOs = Genetically Modified Organisms. ^a^ References refers to the first theorization of the strategy; ^b^ Costs that are common to all strategies can be found in the main text and are omitted here. ^c^ References refer to the most advanced stage of deployment. ^d^ These strategies require repeated releases of sterilizing males and effects drop as releases are stopped unless eradication is achieved; ^e^ In certain species, the doses needed to achieve full male sterility could determine a too high decrease in male mating competitiveness; ^f^ These costs also include those determined by the necessity to transport pupae or adults to be irradiated to the irradiation facility in the case that the radiation source is not managed in the mass rearing facility.
